# Oral Carbon Monoxide Enhances Autophagy Modulation in Prostate, Pancreatic, and Lung Cancers

**DOI:** 10.1002/advs.202308346

**Published:** 2023-12-12

**Authors:** Jianling Bi, Emily Witt, Megan K. McGovern, Arielle B. Cafi, Lauren L. Rosenstock, Anna B. Pearson, Timothy J. Brown, Thomas B. Karasic, Lucas C. Absler, Srija Machkanti, Hannah Boyce, David Gallo, Sarah L. Becker, Keiko Ishida, Joshua Jenkins, Alison Hayward, Alexandra Scheiflinger, Kellie L. Bodeker, Ritesh Kumar, Scott K. Shaw, Salma K. Jabbour, Vitor A. Lira, Michael D. Henry, Michael S. Tift, Leo E. Otterbein, Giovanni Traverso, James D. Byrne

**Affiliations:** ^1^ Department of Radiation Oncology University of Iowa 200 Hawkins Drive Iowa City IA 52242 USA; ^2^ Department of Biomedical Engineering University of Iowa 200 Hawkins Drive Iowa City IA 52242 USA; ^3^ Holden Comprehensive Cancer Center University of Iowa Iowa City IA 52242 USA; ^4^ Department of Biology and Marine Biology University of North Carolina Wilmington Wilmington NC 28403 USA; ^5^ Abramson Cancer Center University of Pennsylvania Philadelphia PA 19146 USA; ^6^ Carver College of Medicine University of Iowa Iowa City IA 52242 USA; ^7^ Department of Chemical Engineering Massachusetts Institute of Technology 25 Ames St. Cambridge MA 02139 USA; ^8^ Department of Surgery Beth Israel Deaconess Medical Center Harvard Medical School 3 Blackfan Circle Boston MA 02215 USA; ^9^ Division of Gastroenterology Brigham and Women's Hospital Harvard Medical School 75 Francis St. Boston MA 02115 USA; ^10^ School of Medicine Oregon Health and Science University Portland OR 97239 USA; ^11^ David H. Koch Institute for Integrative Cancer Research Massachusetts Institute of Technology 500 Main St Building 76 Cambridge MA 02142 USA; ^12^ Department of Mechanical Engineering Massachusetts Institute of Technology 77 Massachusetts Ave Cambridge MA 02139 USA; ^13^ Division of Comparative Medicine Massachusetts Institute of Technology Cambridge MA 02139 USA; ^14^ Department of Radiation Oncology Rutgers Cancer Institute of New Jersey Robert Wood Johnson Medical School New Brunswick NJ 08903 USA; ^15^ Department of Chemistry The University of Iowa Iowa City IA 52242 USA; ^16^ Department of Health & Human Physiology University of Iowa Iowa City IA 52242 USA; ^17^ Department of Molecular Physiology and Biophysics Carver College of Medicine University of Iowa Iowa City IA 52242 USA; ^18^ Simmons Comprehensive Cancer Center University of Texas Southwestern Medical Center Dallas TX 75390 USA

**Keywords:** autophagy modulation, CO biofoams, combination therapies, smoking

## Abstract

Modulation of autophagy, specifically its inhibition, stands to transform the capacity to effectively treat a broad range of cancers. However, the clinical efficacy of autophagy inhibitors has been inconsistent. To delineate clinical and epidemiological features associated with autophagy inhibition and a positive oncological clinical response, a retrospective analysis of patients is conducted treated with hydroxychloroquine, a known autophagy inhibitor. A direct correlation between smoking status and inhibition of autophagy with hydroxychloroquine is identified. Recognizing that smoking is associated with elevated circulating levels of carbon monoxide (CO), it is hypothesized that supplemental CO can amplify autophagy inhibition. A novel, gas‐entrapping material containing CO in a pre‐clinical model is applied and demonstrated that CO can dramatically increase the cytotoxicity of autophagy inhibitors and significantly inhibit the growth of tumors when used in combination. These data support the notion that safe, therapeutic levels of CO can markedly enhance the efficacy of autophagy inhibitors, opening a promising new frontier in the quest to improve cancer therapies.

## Graphical abstract

Carbon monoxide potently induces autophagy in cancer cells through increased autophagosome formation. It is demonstrated that the combination of orally administered carbon monoxide biofoams generates a robust anti‐cancer effect when combined with autophagy inhibitors in vitro and in vivo. This finding is bolstered by actively smoking cancer patients, which is a state of elevated carbon monoxide levels, who received autophagy inhibitors in clinical trials.

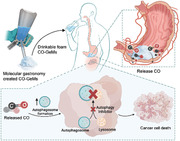

## 1 Introduction

Autophagy is a natural process by which cells degrade and recycle intracellular components. It plays an important role in maintaining cellular health and preventing the accumulation of damaged or dysfunctional organelles and proteins,^[^ ^1^ ^]^ and it can be induced by a variety of conditions including nutrient deprivation, oxidative stress, and cancer cell proliferation.^[^ ^2^ ^]^ In established tumors, enhanced autophagic flux frequently facilitates the survival and proliferation of cancer cells. Thus, modulation of autophagy, especially its inhibition, has been developed for cancer therapy.^[^ ^3^ ^]^

The clinical impact of autophagy inhibitors has been limited because reports of their efficacy have been difficult to interpret.^[^ ^4^ ^]^ For example, in a phase II study of patients with metastatic pancreatic cancer, the addition of an autophagy inhibitor to the chemotherapies gemcitabine and nab‐paclitaxel did not significantly improve overall survival, yet there was a significant improvement in the objective response rate.^[^ ^4j^ ^]^ Interestingly, among the methods that have been proposed to bolster the effectiveness of autophagy inhibitors in treating cancer is the concomitant induction of autophagy.^[^ ^5^ ^]^

Autophagy has been shown to be responsive to gasotransmitters.^[^ ^6^ ^]^ Of the well‐known gasotransmitters, carbon monoxide (CO) has been found to potently induce autophagy in normal cells through increases in mitochondrial reactive oxygen species (ROS).^[^ ^6^, ^7^ ^]^ Recognizing that circulating CO levels are elevated in actively smoking patients we conducted a retrospective analysis of patients treated with the known autophagy inhibitor hydroxychloroquine. We identified smoking status as being directly correlated with a positive oncologic response to hydroxychloroquine. Given this observation, we hypothesized that exogenous CO could enhance the anti‐cancer effect of autophagy inhibitors when administered in combination. To test this hypothesis, we developed a method for CO dosing that is simple and translatable. Dosing of CO by inhalation has unique challenges including high variability in patient ventilation and environmental safety issues such as the need for large, compressed CO gas cylinders. Taking inspiration from the burgeoning culinary field of molecular gastronomy, we circumvented these issues by creating gas‐entrapping materials (GeMs) for the therapeutic oral delivery of CO.^[^ ^8^ ^]^ These materials offer robust and safe oral delivery of CO, using simple, cost‐effective, and off‐the‐shelf components.^[^ ^8^, ^9^ ^]^ In this formulation, CO is readily diffusible through the gastrointestinal (GI) tract, providing systemic exposure for easy administration in combination with other treatments.^[^ ^8^, ^10^ ^]^

Here, we provide clinical data showing an association between smoking, a state of elevated carbon monoxide exposure, and clinical response to autophagy inhibitors in pancreatic and lung cancer patients. We then report on our development of robust, orally administered CO‐GeMs that enable consistent systemic CO exposure in small and large animals. In our analysis of multiple cancer cell lines, including prostate, pancreatic, and lung cancer, CO induced autophagy and the use of exogenous CO significantly increased the cytotoxicity of several autophagy inhibitors. In mouse models of cancer, concomitant treatment with oral CO‐GeMs plus autophagy inhibitors had profound synergistic anti‐cancer effects noted by tumor growth inhibition and immunohistochemical analysis.

## 2 Results

### 2.1 Active Smokers have Excellent Response to Autophagy Inhibitors in Clinical Trials

**Figure 1:**
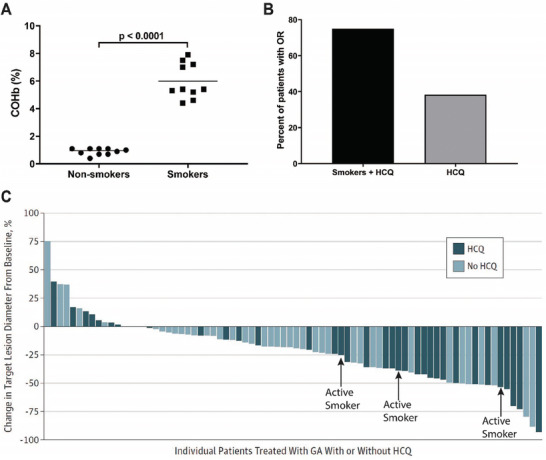
Active smokers treated with autophagy inhibitors had excellent outcomes during clinical trial. A) COHb % in whole blood measured using a blood gas analyzer of non‐smokers versus active smokers within 3 h of their last cigarette (*n* = 10 per arm, *p* < 0.0001). *P* values were determined by unpaired *t* test. B) Intention‐to‐treat analysis for overall response (OR) in active smokers versus all patients. C) Change in diameter of target lesions from baseline, with active smokers identified by each arrow. Subfigure (C) was modified with permission from JAMA Oncology.

To motivate the concomitant use of autophagy inducers with autophagy inhibitors, we identified individuals within clinical trials of autophagy inhibitors that were in a state of increased autophagy. Active smokers are known to have increased cellular autophagy, resulting from CO and other excipient exposure. To validate that smokers have elevated carboxyhemoglobin (COHb), we assessed COHb in healthy individuals that recently smoked a cigarette (0–3 h prior). As expected, COHb was significantly greater in those actively smoking versus non‐smokers with an average COHb of 5.9 compared to 0.7 in non‐smokers (**Figure** **1**A). We next evaluated the impact of active smoking on the effectiveness of autophagy inhibitors based on retrospective analysis of data from clinical trials (ClinicalTrials.gov identifiers: NCT01506973, NCT00728845, and NCT01649947). Eighteen clinical trials have assessed the utility of autophagy inhibitors, alone or in combination with chemotherapies or other agents, in treating cancer patients (Table S1, Supporting Information).^[^ ^4^ ^]^ We retrospectively analyzed data from two of these trials, specifically those involving pancreatic and lung cancer patients.^[^ ^4j,l^ ^]^ Smoking status was verified through the electronic medical record and outcomes were assessed based upon smoking status. Among the pancreatic cancer patients who received the autophagy inhibitor, hydroxychloroquine (HCQ), 4 out of 42 patients were found to have actively smoked during the study; out of the 4 individuals, 1 patient was non‐evaluable due to early dropout. In addition, overall response of patients to autophagy inhibitors was greater in individuals who actively smoked while on trial compared to the entire group (Figure 1B; Figure S1A, Supporting Information). The percent reduction in size of the target lesion was excellent in individuals who actively smoked on trial (Figure 1C); a similar effect was noted in lung cancer patients receiving HCQ (Figure S1B, Supporting Information).

### 2.2 CO‐GeMs Design and Bioavailability

**Figure 2:**
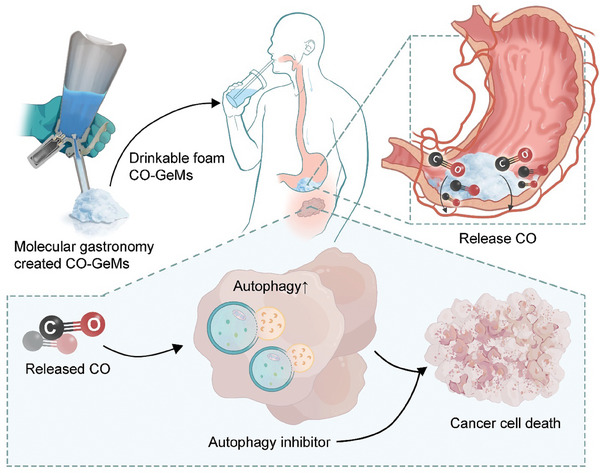
Schematic illustrating how CO‐GeMs are administered in combination with autophagy inhibitors and how they reach their targets. CO induces autophagy and when combined with downstream autophagy inhibition results in enhanced cancer cell death.

**Figure 3:**
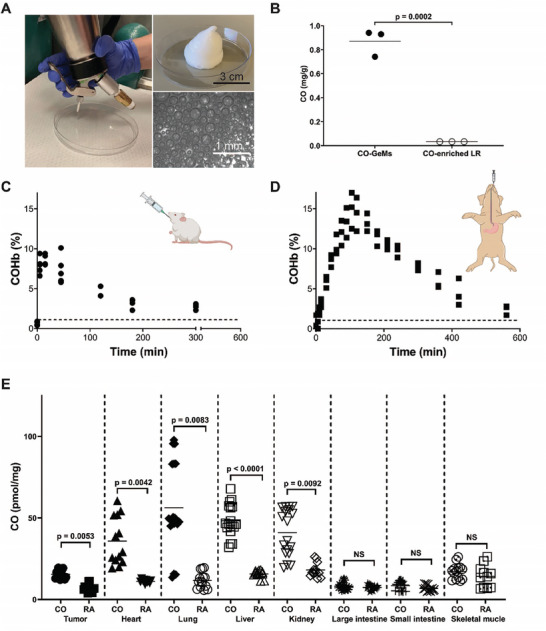
CO‐GeMs achieve sustained, elevated COHb in both small and large animals. A) Pressurized vessel for creation of CO‐GeMs and macroscopic and microscopic images of CO‐GeMs. B) Concentration of CO delivered by GeMs and CO‐enriched lactated Ringer's solution (LR). Percentages of COHb measured in C) mice and D) pigs at the indicated time after oral CO‐GeM administration (*n* = 5 per group). The dotted line represents highest baseline COHb values. E) Organ‐specific concentrations of CO in mice at 15 min after CO‐GeM administration via oral gavage (5 g kg^−1^). Data are means (*n* = 5 animals per arm, with each sample evaluated in triplicate). *P* values were determined by unpaired *t* test comparing tissue CO concentration between animals that received oral CO‐GeMs (CO) versus animals that received oral GeMs infused with room air (RA).

To generate the CO‐GeMs, commercially available whipping siphons were used to physically entrap CO in Generally Recognized as Safe (GRAS) materials. **Figure** **2** shows by schematic how CO‐GeMs are administered and how they reach their target tissues, as well as their synergistic anti‐cancer effects when used in combination with autophagy inhibitors. These pressurized vessels were reverse engineered to introduce specific concentrations of gases within a GeM matrix, as reported previously.^[^ ^8^ ^]^ A custom‐made connector facilitated pressurization with any gas, and a one‐way valve was incorporated to maintain gas pressure. **Figure** **3**A shows the whipping siphon and CO‐GeMs generated from it, at the macroscopic and microscopic levels.

The CO‐GeMs were next tested for the efficiency of gas entrapment using Fourier Transform Infrared (FTIR) spectroscopy and gas chromatography. These materials encapsulated approximately 26 times more CO than previously published CO‐enriched materials (Figure S2, Supporting Information; Figure 3B, CO‐GeMs versus CO‐enriched Lactated Ringer's (LR)). The total percentage of CO by volume in the CO‐GeMs was ≈90 ± 5%. Furthermore, bubble size in each CO‐GeM was noted to increase over time (Figure S3, Supporting Information).

The volumetric stability of the GeM foams within biological samples could be enhanced by increasing the concentration of the thickening agent (xanthan gum), as was evaluated in synthetic gastric fluid (Figure S4, Supporting Information). The CO‐GeMs were stable in gastric fluid for several hours (Figure S4, Supporting Information) and were found to release all CO by 24 hours (Figure S5, Supporting Information). Notably, earlier rheological analysis of CO‐GeMs showed that they can easily flow through a straw or syringe,^[^ ^8^ ^]^ facilitating their administration.

### 2.3 Delivery of CO by GeMs was Effective in Small and Large Animals

The pharmacodynamics of CO delivered through oral administration of CO‐GeMs were characterized in both small and large mammals (Figure 3C,D). In mice, COHb reached a peak of 8.7 ± 0.7% at 15 min after a single oral dose of the CO‐GeM was administered and decreased over six hours with a t_1/2_ of 136 min. In pigs, COHb reached a peak of 14.9 ± 2.3% at 1.75 h and decreased over ten hours after a single oral dose; t_1/2_ of 5.17 h.

We further evaluated the pharmacokinetics of the orally administered CO‐GeMs by using a near IR‐dye‐labeled xanthan gum, which demonstrated the xanthan gum remained exclusively in the GI tract (Figure S6, Supporting Information). The concentration of CO was then measured in various tissue compartments in mice bearing subcutaneous syngeneic prostate tumors following administration of CO‐GeM (Figure 3E). Significantly more CO was detected in the tumor and other tissues 15 min after administration, particularly in the liver, heart, spleen, lungs, and kidney, compared to room air (RA)‐GeM‐treated mice. However, the small and large intestines of these animals were not found to have higher CO amounts than that in RA‐GeM‐treated controls.

### 2.4 Exogenous CO Induces Autophagy in Cultured Cancer Cells

**Figure 4:**
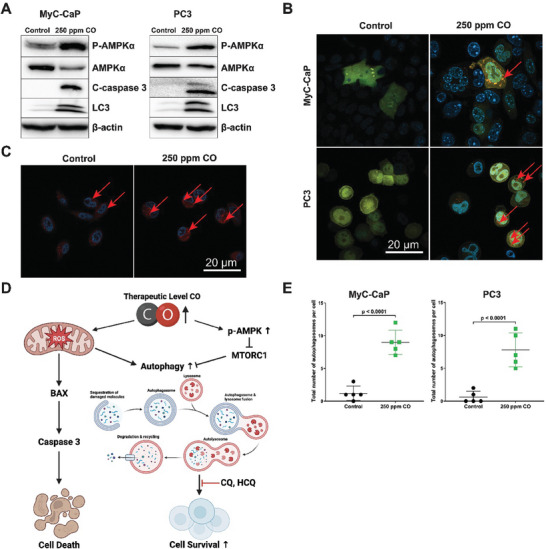
CO induces autophagy in cancer cells. A) Western blotting to assess levels of phosphorylated‐AMPKα, LC3, and cleaved caspase 3 in mouse prostate cancer cells (MyC‐CaP, left) and (PC3, right) exposed to CO or room air. Right: Quantification of Western blotting results for the same proteins in human prostate cancer cells (PC3). Western blots are representative images from 3 independent experiments. B) Autophagic flux in cells transduced with an adenovirus coding for GFP‐RFP‐LC3 CO versus room air. Red arrows indicate autophagosomes. C) Expression of the lysosomal marker LAMP2 in MyC‐CaP cells exposed to CO or room air. D) Proposed model for how CO exposure induces autophagy and the impact of combined autophagy inhibition. We show that exogenous CO increases phosphorylation of AMPK and mitochondrial ROS as previously shown (*23*). Both mechanisms lead to an increase in autophagy. E) Quantification of autophagosomes from subfigure B. P values were determined by one‐way ANOVA. Results represent mean ± SD of 3 independent experiments.

To characterize the induction of autophagy by CO, human and mouse prostate, pancreatic, and lung cancer cell lines were exposed to CO dose (250 ppm) (**Figure 4**; Figure S7, Supporting Information). Cells exposed to CO had higher levels of phosphorylated‐AMPKα (p‐AMPKα) and LC3, both markers of autophagy (Figure 4A), as well as a higher LC3BII/I ratio (Figure S8, Supporting Information).

Autophagic flux is a dynamic process, and it was critical to distinguish between increased formation and decreased clearance of autophagosomes. To this end, we infected PC3 and MyC‐CaP cells with an adenovirus GFP‐RFP‐LC3 before exposure to CO (250 ppm).^[^ ^11^ ^]^ This construct serves as a reporter of autophagic flux because in transduced cells, the GFP fluorescence is quenched in the acidic lysosomal compartment, yet RFP continues to fluoresce and marks both autophagosomes and autolysosomes. Thus, merged images in yellow represent autophagosomes that can be evaluated while red represent autolysosomes.^[^ ^11b^ ^]^ In the presence of CO, the number of autophagosomes identified by confocal microscopy was significantly higher than compared to untreated (air) controls (Figure 4B,E).

Next, we performed immunofluorescence staining for lysosomal membrane protein 2 (LAMP2), an important regulator of autophagy.^[^ ^12^ ^]^ In MyC‐CaP cells exposed to CO (250 ppm), LAMP2 content was higher than air controls not exposed to CO (Figure 4C). In the context of past work by others,^[^ ^6c^ ^]^ these results suggest that autophagic induction by CO is driven largely through increases in mitochondrial ROS and p‐AMPKα (Figure 4D).

### 2.5 CO Enhanced Anti‐Cancer Effects of Autophagy Inhibitors

**Figure 5:**
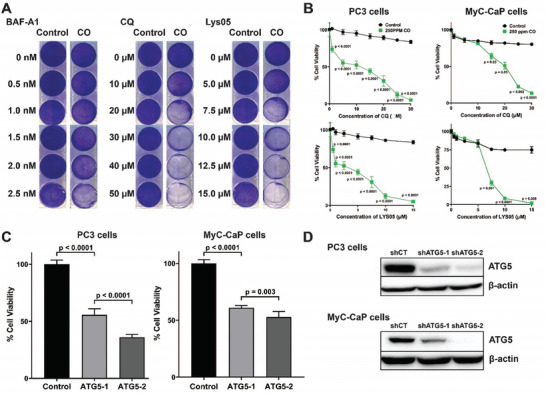
Co‐administration of CO and autophagy inhibitors are synergistic in cancer cell death. A) Cytotoxicity, as determined by crystal violet staining, of MyC‐CaP cells exposed to increasing doses of the autophagy inhibitors chloroquine (CQ) and Lys05 ± CO (250 ppm). Control groups were exposed only to the inhibitors in standard incubator conditions (5%CO_2_) without CO. B) Quantification of the viability (IC_50_ values) of PC3 and MyC‐CaP cells as a function of CQ and Lys05 dose. C) Quantification of cell viability for PC3 and MyC‐CaP cells subjected to shATG‐mediated knockdown of ATG5. Controls were non‐transduced cells exposed to 250 ppm CO. D) Western blotting showing levels of ATG5 protein in the cells shown in C. P values were determined by one‐way ANOVA. Results represent mean ± SD of 3 independent experiments. Western blots are representative of 3 independent experiments.

To test our hypothesis that concomitant administration of exogenous CO can enhance the anti‐cancer effect of autophagy inhibitors, we performed in vitro cell survival assays. Crystal violet staining of MyC‐CaP cells exposed to increasing doses of the autophagy inhibitors bafilomycin A1 (BAF‐A1), chloroquine (CQ), and Lys05 demonstrated that the cytotoxicity of the inhibitors was increased in the presence of CO (250 ppm), compared to treatment with either CO (250 ppm) or inhibitor alone (**Figure** **5**A). Notably, CO had no effect on cell viability in the absence of the autophagy inhibitor in cancer cells and normal human primary intestinal cells (Figure S9, Supporting Information), suggesting that CO has minimal cytotoxicity. To validate the specificity of the effect of CO on cell viability, IC_50_ values for these autophagy inhibitors, in the presence and absence of CO, were measured using the alamarBlue assay. IC_50_ values for multiple autophagy inhibitors were significantly reduced in both prostate (MyC‐CaP and PC3), pancreatic (Panc02 and MIA PaCa‐2), and lung (A549) cancer cells exposed to CO (Figure 5B; Figure S10, Supporting Information, respectively). To determine whether this effect involved classic autophagy, shATG lentivirus was used to knock down ATG5 and ATG7 in PC3 and MyC‐CaP cells. In these cells, CO exposure without autophagy inhibitors significantly reduced cell viability compared to room air control (Figure 5C). The expression levels of knocked down ATG5 and 7 are shown in Figure 5D, Figures S11 and S12 (Supporting Information).

### 2.6 Co‐Administration of CO‐GeMs and Autophagy Inhibitors Suppressed Tumor Growth in Mice

**Figure 6:**
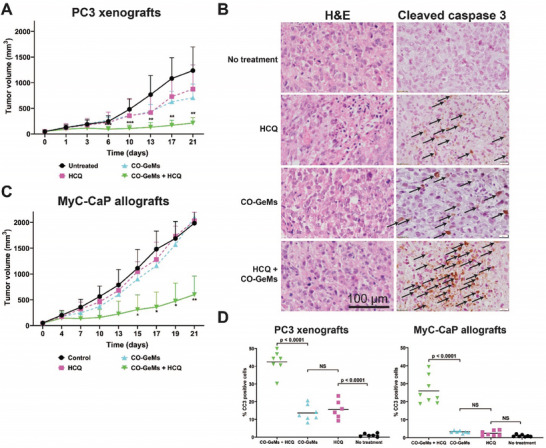
Anti‐tumor effects of CO‐GeMs and hydroxychloroquine (HCQ) are enhanced when they are combined. A) Tumor volume in human prostate cancer xenografts treated with CO‐GeMs and HCQ, alone and in combination. Result represent mean± SD of 7 mice/group B) Histological staining of representative tumors in (A) for H&E and cleaved caspase 3. C) Quantification of tumor volume in murine syngeneic model of prostate cancer following treatment with CO‐GeMs and HCQ, alone and in combination. D) Quantification of cleaved caspase 3‐positive areas analyzed in randomly selected 600 µm x 600 µm sections of tissue at full thickness t (×8 magnification). Data represent means (*n* = 7 mice/group, three replicates per mouse). P values were determined by one‐way ANOVA. * – *p* < 0.05; ** – *p* < 0.01.

Given the demonstrated benefit of co‐administration of CO plus autophagy inhibitors in vitro, we next evaluated the effectiveness of this strategy using CO‐GeMs in mouse models of prostate (PC3 xenografts and MyC‐CaP allografts) and pancreatic cancer (Panc02 allografts). For each model, four treatment groups were assessed: CO‐GeM + HCQ; CO‐GeM alone; HCQ alone; and no treatment. In both models, once a tumor reached 50–100 mm^3^ in volume, the mice were treated with the relevant formulation by oral gavage. The greatest reduction in tumor growth was observed in mice receiving the CO‐GeM + HCQ (p < 0.01) by 21 days (**Figure** **6**A,C; Figure S13, Supporting Information). In the PC3 xenografts, there was some benefit from the individual treatments, but not nearly as marked as observed with the co‐treatment. This effect on tumor size is supported by staining for cleaved caspase 3 (CC3), a marker of apoptosis (Figure 6B,D), which revealed significantly greater staining in the CO‐GeM + HCQ arms compared to all others (*p* < 0.0001). The induction of autophagy was confirmed in the MyC‐CaP allograft tumors after exposure to CO‐GeM (Figure S14, Supporting Information). Weights of the mice were stable throughout treatment and alanine transaminase (ALT) values were unchanged in mice treated with CO GeMs, as shown in Figures S15 and S16 (Supporting Information), suggesting safety of this combination treatment.

## 3 Discussion

Enthusiasm for the use of autophagy inhibition as a component of cancer therapy has been dampened by mixed results of clinical trials.^[^ ^4^ ^]^ Notably, of the published clinical trials involving autophagy inhibitors to date, only one randomized phase II trial has provided evidence of improvement in clinical endpoints when the inhibitor arm was compared to the control (Table S1, Supporting Information).^[^ ^4j^ ^]^ One new strategy that has been proposed for improving this treatment response is to trigger autophagy and concomitantly block a downstream step of the pathway, such as fusion of autophagosomes with lysosomes.^[^ ^5^ ^]^

To motivate this strategy, we retrospectively evaluated prospectively collected data from different clinical institutions, which revealed that autophagy inhibitors are particularly effective in active smokers. Smokers have been shown here to have elevated systemic CO levels as measured by blood COHb compared to non‐smokers. Importantly, other excipients in cigarettes have been shown to promote autophagy in active smokers, a result that could potentially contribute to our positive response with HCQ.^[^ ^13^ ^]^ In addition, we demonstrate that autophagy of cancer cells can be induced by exposure to exogenous CO. This autophagy induction appears to be mediated by CO‐dependent increases in mitochondrial ROS levels, which lead to AMPK activation.^[^ ^6c^ ^]^ We further found that when CO treatment was combined with autophagy inhibitors (e.g., HCQ), a significant anti‐cancer effect occurred both in vitro and in vivo in both prostate and pancreatic cancer models. These efforts are certainly not to encourage smoking but to showcase a potential therapeutic advantage to exploit through exogenous CO administration.

Other studies have demonstrated that a low dose of CO alone, administered via inhalation, has an anti‐cancer benefit, as does the use of CO‐releasing molecules.^[^ ^14^ ^]^ CO has also been shown to lead to an anti‐Warburg effect, causing a metabolic shift from anaerobic glycolysis to oxidative phosphorylation. In these conditions, decreased rates of adenosine triphosphate resynthesis can lead to energy stress which stimulates autophagy.^[^ ^15^ ^]^ Additionally, inhaled CO also synergized with doxorubicin and camptothecin which collective with the data presented here may allow for chemosparing treatment regimes, thus decreasing toxicity. We used a logical strategy to combine CO with autophagy inhibitors and found that the combination resulted in a synergistic anti‐cancer effect.

The translatability of our findings to therapies for cancer patients will depend on methods for CO delivery and safety of the materials. Numerous completed and ongoing clinical trials have previously shown or are currently investigating the use of inhaled CO. All trials completed to date have concluded that CO treatment is extremely safe, especially in immunocompromised patients such as those who have interstitial pulmonary fibrosis.^[^ ^16^ ^]^ Acceptability by physicians might be enhanced if a convenient method for dosing of CO, such as the use of CO‐GeMs, is proposed versus having to use cumbersome inhalational delivery. Additionally, an orally active agent lends itself to administration inside and outside of a hospital setting. The whipping siphon can generate discrete doses of orally active agents, which can enable safe administration. Moreover, the materials used in this study were found to be safe, biocompatible, and well‐tolerated as demonstrated by animal weight stability and liver function tests.

Improvements in dosing of CO‐GeMs will require additional work on formulations in healthy patients. In particular, it will be important to identify doses that lead to COHb levels that fall under the FDA limit of 14%. The feasibility of this approach will be better understood once additional knowledge of the pharmacokinetics of CO administered via GeMs is determined, especially with respect to resulting intratumoral concentrations. The pre‐clinical work described herein used subcutaneous models of cancer, in which perfusion is known to be less efficient than in orthotopic tumors.^[^ ^17^ ^]^ Thus, we expect that GeMs will allow for larger quantities of CO to be delivered to orthotopic or primary tumors. Even though the clinical trials evaluated support the translational implications of our pre‐clinical studies, a couple of limitations should be considered: a) data was retrospective; b) sample sizes were small; and c) smoking likely impacts autophagy via CO‐independent mechanisms as well. Moreover, the amount of CO in the body was not measured during treatment thus it may be that a prophylactic treatment is sufficient to observe synergistic effects with other treatment strategies. Ultimately, the clinical use of a combination treatment regimen with CO‐GeMs and autophagy inhibitors will still require prospective studies.

## 4 Conclusions

In summary, the strategy we propose herein has the potential to provide a substantial benefit for cancer patients through its unique modulation of autophagy. Moreover, given that the combination of CO with autophagy inhibitors was effective in treating both prostate and pancreatic cancers in our model systems, it will likely be applicable to a wide variety of malignancies. Additionally, the anti‐inflammatory properties of CO may make this approach well suited for use with therapies that typically lead to tissue injury, such as radiation therapy.^[^ ^8^ ^]^ Of note, the GeM strategy may potentially be extended to concomitantly deliver other agents and be useful for other combination therapies.

## 5 Experimental Section

### Study Design

The aim of this study was to evaluate the anti‐cancer impact of CO when used in combination with autophagy inhibitors. Safe, low‐cost materials were used to enable oral delivery of CO. The GeMs were produced using pressurized systems, and their pharmacodynamics and pharmacokinetics were analyzed in healthy mice and swine. First, the in vitro impact of combination CO + autophagy inhibitors was determined, and then the in vivo efficacy of CO‐GeMs + autophagy inhibitors was determined in subcutaneous syngeneic (MyC‐CaP) and xenograft (PC3) prostate cancer models, as well as in a subcutaneous syngeneic (Panc02) pancreatic cancer model (5‐8 mice in each arm). The Institutional Animal Care and Use Committees at the Massachusetts Institute of Technology (0519‐023‐22) and the University of Iowa (1092429‐008) approved the use of animals and the proposed protocols. The pathologist was blinded to study arms before and during histological analysis; the investigators and animal technicians were not blinded. All animals were included in the analysis.

### Retrospective Analysis of Clinical Trials

Retrospective analysis was conducted on samples obtained from NCT01506973, NCT00728845, and NCT01649947 for whom participants provided written prospective consent to share data and biospecimens; a data usage agreement was fully executed between institutions. Active smokers among the clinical trial patients were identified by clear documentation of active smoking in the electronic medical record. The Institutional Review Boards at the University of Pennsylvania (#814 704), HonorHealth #814 704), the Johns Hopkins University (#814 704) and Rutgers (#022 011 0249). The clinical trials were conducted in accordance with the ICH Good Clinical Practice E6 as adopted by the US Food and Drug Administration.^[^ ^4j^ ^]^

### Assessment of COHb from Smokers Versus Non‐Smokers

The human subject's study was approved by the University of Iowa Institutional Review Board (IRB 292 393 443). Informed consent was obtained prior to collection of blood samples from healthy volunteers – smokers and non‐smokers. Volunteers were recruited by emailing the UIowa listserv, and one 4 mL vial of venous blood was obtained from each volunteer in a heparinized tube. In smokers, blood was obtained 0–3 h after smoking a cigarette. The samples were evaluated for COHb using ABL80 FLEX CO‐OX blood gas analyzer.

### Formulation Development

The pre‐foam GeM solution was prepared by dissolving 0.5 wt.% xanthan gum (Modernist Pantry) and 0.8 wt.% methylcellulose (Modernist Pantry) in distilled, deionized water. This solution was then heated to 100°C while being stirred at 700 rotations per minute and was later cooled to room temperature. It was then degassed under a vacuum for 12 h. A modified iSi 1 Pint 100 stainless steel whipping siphon was used, with a custom‐made aluminum connector that allowed for pressurization using CO and medical‐grade air gas cylinders. The aluminum connector used to connect the whipping siphon to the gas regulator was created using a lathe to cut the part to size, and an M22 tap and 1/4 NPT die to form threads in the connector. The whipping siphon was purged with CO (99.3%) pressurized to 200 PSI to create the CO‐GeMs or purged with medical air (21% O_2_ with 0% CO; Linde) to create the room air GeMs (RA‐GeMs), shaken for 30 seconds, and then dispensed into a syringe.

### Material Characterization

Initially, the materials were studied and characterized both macroscopically and microscopically. An EVOS microscope (10X magnification) enabled microscopic evaluation of the gas bubbles in CO‐GeMs. The distribution of bubble size was determined by placing 1 mL of foam into a 24‐well plate and performing serial microscopy at designated times. The volumetric stability of the foams was determined by placing 100 mL samples into a 250 mL graduated cylinder pre‐filled with 10 mL of synthetic gastric fluid (Carolina Biological), maintaining them in a humidified chamber at 37°C, and recording the foam volume and liquid volume fractions at designated times, based on visual inspection.

To quantify CO in the materials, an Agilent gas chromatograph‐thermal conductivity detector (GC‐TCD) with helium as the carrier gas was used. CO‐GeM samples underwent three vacuum‐nitrogen (99.9%) purge cycles before use. The samples were subsequently placed into borosilicate glass GC vials and shaken at 37°C for 48 h to release CO completely. Each sample was then run in triplicate on the GC‐TCD, with calibration curves generated using the 99.3% CO cylinders that had been used to generate the GO‐GeMs. FTIR spectroscopy was performed as additional characterization to demonstrate CO entrapment. Infrared data are acquired using a Thermo iS50 FTIR spectrometer. In each of the two trials shown, 50 µL of the foam was gently pressed between two 1.5 mm thick CaF2 salt plates. Absorption spectra of this “sandwich cell” are collected in a standard transmission geometry at 2 cm‐1 resolution. Spectra for trial 1 and trial 2 represent averages of 16 interferograms and are each refenced to independent background spectra for the same salt plates, with no foam, held in standard room air. Bubble size analysis was performed using Image J, where 10 images were analyzed per time point for each formulation.

### In Vitro Studies—Western Blotting

Cells were collected and lysed with RIPA buffer (sc‐24948, Santa Cruz Biotechnology). Equal amounts of protein (40 µg) were separated on 10% or 12% SDS‐polyacrylamide gels and then transferred to a nitrocellulose blotting membrane (PALL Corporation, Mexico) or a PVDF blotting membrane (Bio‐Rad Laboratories, Hercules, CA, USA). After blocking with 5% non‐fat milk, the membrane was incubated overnight with the respective primary antibody at 4 °C. The following antibodies were obtained from Cell Signaling (Danvers, MA, USA): anti‐LC3B (1:1000, #83 506), anti‐ATG5 (1:1000, #12 994), anti‐ATG7 (1:1000, #2631), anti‐pAMPKα (1:1000, #2535), anti‐AMPKα (1:1000, #5832), anti‐cleaved caspase 3 (1:1000, #9661). The control anti‐β‐actin (1:10000, sc‐47778) was obtained from Santa Cruz Biotechnology (Dallas, TX, USA). Signal was detected using the Bio‐Rad ChemiDoc system and signal density was measured using the Bio‐Rad Image Lab Software (Bio‐Rad Laboratories, Redmond, WA, USA).

### In Vitro Studies—Cell Viability Assays

MyC‐CaP, PC3, Panc02, MIA‐PaCa‐2, and A549 cells were seeded into 96‐well plates (10 000 cells per well) for 24 h, treated with increasing concentrations of autophagy inhibitors in a sealed exposure chamber (StemCell Technologies) with 250 parts per million (ppm) CO mixed with 5% CO_2_ balanced air for an additional 72 h. In a separate experiment, FHs 74 Int cells were seeded into 96‐well plates (10 000 cells per well) for 24 h and exposed 250 parts per million (ppm) CO mixed with 5% CO2 balanced air for an additional 72 h. Cell viability was evaluated using the cell proliferation reagent alamarBlue (ThermoFisher Scientific) according to the manufacturer's protocol. The absorbance was measured using a micro‐plate reader (Bio‐Rad Laboratories, Inc., Hercules, CA, USA) with a 560/590 nm (excitation/emission) filter. All experiments included three technical replicates. Data were normalized to untreated control, set at 100% viability.

### In Vitro Studies—shRNA‐Mediated Knockdown of ATG5 and ATG7

Low passage number HEK293T cells were transfected with the PLKO.1 vector containing a nontargeting shRNA or shRNAs against mouse and human ATG5 (TRCN0000099432 and TRCN0000375819, TRCN0000330394 and TRCN0000151474 obtained from the RNAi Consortium) and ATG7 (TRCN0000007584 and TRCN0000007587), along with PAX2 and VSVG, using Lipofectamine 2000 Reagent according to the manufacturer's protocol. Lentivirus particles were collected 48 hours post transfection, supplemented with 10 µg mL^−1^ polybrene, and stored at −80°C. Six‐well plates were seeded with 1 × 10^5^ MyC‐CaP and 1 × 10^5^ PC3 cells, after which lentivirus was added for 24 h. Cells were then selected for puromycin resistance (6 µg mL^−1^ and 3 µg mL^−1^) for 7 days. The efficiency of ATG5 and ATG7 knockdown in cells was determined by Western blotting, as listed above.

### In Vitro Studies—Crystal Violet Staining

Six‐well plates were seeded with 1 × 10^5^ MyC‐CaP cells and 1.3 × 10^5^ PC3 cells for 24 h. Autophagy inhibitors (BAF‐A1, Lys05, CQ) were added to the media of cells at the concentrations listed. Subsequently, the cells were exposed to 250 ppm CO in hypoxia chambers for 48 h. The media was then removed, cells were washed with cold PBS, fixed, stained with 0.5% crystal violet solution for 30 min, and then rinsed with DI water.

### In Vitro Studies—IHC Staining and Analysis

Sections of 4% paraformaldehyde‐fixed paraffin‐embedded tissue samples, cut at 5‐µm thickness, were used for immunostaining. Staining for cleaved caspase 3 was performed on paraffin sections using a rabbit anti‐cleaved caspase 3 antibody (1:200; Cell Signaling). The secondary antibody was a biotinylated goat anti‐rabbit IgG (1:200; BA‐1000; Vector Laboratories). Staining was performed using the Vector ABC kit and the Vector DAB kit (Vector Laboratories). Staining for LC3BII was performed on paraffin sections using a rabbit polyclonal LC3B antibody (1:200; Novus Biologicals). ImmPRESS HRP Goat Anti‐Rabbit IgG Polymer Kit (Vector Laboratories) was used as a secondary antibody. Staining was performed using a DAB substrate kit (Vector Laboratories).

### In Vitro Studies— Adenovirus Transduction

Adenovirus‐RFP‐GFP‐LC3 was kindly provided by Dr. Ling Yang (University of Iowa). PC3 and MyC‐CaP cells were transduced with adenovirus‐RFP‐GFP‐LC3 at a titer of 10 MOI for 24 h, then incubated with 250 ppm CO for 30 h. Cells were then fixed and processed for immunofluorescence staining. Visualization was performed on a Zeiss 980 confocal microscope.

### In Vitro Studies—Immunofluorescence Imaging

Coverslips were seeded with 1 × 10^5^ PC3 cells for 24 h, then incubated with 250 ppm CO for 30 h. Coverslips were rinsed with PBS then fixed with 2% paraformaldehyde for 20 min, followed by permeabilization for 30 min with 0.2% Triton X‐100. Cells were blocked with 3% BSA and then incubated with anti‐LAMP2 (1:50, DSHB GAM‐568) at 4 °C overnight. Cells were then incubated with Alexa Fluor 546‐conjugated anti‐rabbit secondary antibody (1:200, Cell Signaling Technology) at room temperature for 2 h; nuclei were stained using mounting solution with DAPI (Vector Laboratories). Visualization was performed on a Zeiss 980 confocal microscope.

### Animal Studies

For rodent studies (*n* = 6–8 per arm), male nude (nu/nu) mice (for the PC3 xenograft model), male FVB mice (for the MyC‐CaP syngeneic model), and female C57BL6/J mice (for the Panc02 syngeneic model) aged 6–8 weeks from Jackson Labs were used. The experiments were conducted in animal facilities at the University of Iowa, after allowing rodents to acclimate to this environment for at least 72 h. For swine studies (*n* = 3), healthy female Yorkshire pigs weighing 65–80 kg were used. These experiments were conducted in the animal facility at Massachusetts Institute of Technology, after allowing swine to acclimate to this environment for at least 7 days. Except where otherwise designated, animals were provided with food and water ad libitum and exposed to a 12‐hour light/dark cycle.

### Animal Studies—Evaluation of Pharmacokinetics and Pharmacodynamics of CO

The pharmacodynamics of CO administered by oral gavage of CO‐GeM was evaluated in mice and swine. Conscious mice were treated with 200 µL CO‐GeM containing 0.106 µg CO and blood samples were obtained through submandibular bleeds into a 150 µL heparinized borosilicate glass hematocrit tube and then capped and stored at −80°C until analysis. Isoflurane‐anesthetized swine were treated with 600 mL CO‐GeM containing 0.318 mg CO through an orogastric tube and blood was collected from an ear vein catheter. The blood was placed into 1‐mL BD syringes filled with 100 units of heparin and analyzed using a Radiometer ABL80 FLEX CO‐OX blood gas analyzer.

For pharmacokinetic analyses, FVB mice‐bearing subcutaneous MyC‐CaP tumors (≈150 mm^3^) were treated with 200 µL CO‐GeM or RA‐GeM p.o. and 15 min later total body perfusion was performed by blood severing the femoral artery and injecting ice‐cold PBS into the heart. The organs were subsequently rinsed with PBS, collected, and snap frozen for analysis. Stool in the small and large intestines were removed and rinsed with PBS before snap freezing. The extraction of CO and its quantification in tissues and blood followed previously established methods.^[^ ^8^, ^18^ ^]^ Mouse tissues were thawed and rinsed with chilled KH_2_PO_4_ buffer (pH 7.4) to remove any remaining excess blood and then placed into microcentrifuge tubes. Ice‐cold de‐ionized water was added to tissues samples at a (10% weight/weight ratio). With the tube on ice, the tissues were cut into smaller pieces using surgical scissors and thoroughly homogenized using an Ultra‐Turrax T8 grinder and ultrasonic cell disruptor. Then 10 µL of the resulting tissue homogenate and 20 µL of 20% sulfosalicylic acid were added to purged amber chromatography vials to release CO into the headspace of the vial. Samples were stored on ice for at least 15 minutes before CO analysis. A CO‐free carrier gas (Ultra‐zero air; AirGas) was then used to push the headspace gas into the reducing compound photometer GC system (GC Reducing Compound Photometer, Peak Performer 1, Peak Laboratories LLC). Standard curves were generated daily for a certified calibration gas (1.02 ppm CO balanced with nitrogen: AirGas). The tissue CO concentrations were reported as pmol of CO per milligram wet weight tissue. For pharmacokinetic evaluation of CO‐GeMs, xanthan gum (Fisher Scientific) was labeled with CF Dye using the CF Dye Aminooxy Kit from Biotium. To start, a 5 mm CF aminooxy stock solution was prepared in DI water. Xanthan gum was prepared in 1X PBS to a final concentration of 50 mm. The protocol for glycoprotein oxidation was followed (1/10 volume 10X reaction buffer (1 m sodium acetate, 1.5 m NaCl in DI water, pH 5.5) and 1/10 volume 100 mm sodium periodate solution in 1X PBS were added to 50 mm xanthan gum solution. The mixture was incubated for 10 minutes at room temperature, and ethylene glycol was introduced to a final concentration of 100 mm to stop the periodate reaction. The sample was further incubated for 10 min at room temperature. Next, 50 molar equivalents of CF aminooxy reagent and 1/10 volume aniline acetate catalyst were added to the xanthan gum solution. The solution was vortexed, and the reaction proceeded in the dark with agitation for two hours at room temperature. The CF‐labeled xanthan gum was purified by centrifugal filter (Amicon Ultra, 10000 MWCO) and then stored at −20°C until ready for use. 10 mL of 1.0 wt.% CF‐labeled xanthan gum was spiked into 50 mL of the 0.5 wt.% xanthan gum formulation, and the whipping siphon was pressurized to 200 mmHg with CO. 200 µL oral gavage of foam was administered to healthy C57BL/6J mice. The animals were euthanized, and organs harvested prior to analysis on an in vivo imager.

### Animal Studies—Efficacy Studies

*Prostate Cancer Mouse Models*: For PC3 xenograft models, 6‐8‐week‐old male nude (nu/nu) mice underwent subcutaneous injection of 1.0 × 10^6^ PC3 cells into the flank. The mice were randomized into different treatment groups (*n* = 7–8 per group) upon reaching between 50–100 mm^3^, including treatment with HCQ (60 mg kg^−1^ intraperitoneal (IP)) + 200 µL CO‐GeMs ter in die (TID)); HCQ (60 mg kg^−1^ IP); 200 µL CO‐GeMs TID; and no treatment. Mice were monitored daily for tumor development, and once a tumor reached a volume of at least 100 mm^3^ (Day 1), the mice were dosed daily for 21 days. Subcutaneous flank tumors were measured three times weekly using calipers. Tumor volume was approximated using the formula V = length (L) × width (W) × width (W)/2, with L and W corresponding to the x and y measurements of the tumor in mm. For the MyC‐CaP syngeneic model, 6–8‐week‐old male FVB mice underwent subcutaneous injection of 3.0 × 10^6^ MyC‐CaP cells into the flank. The mice were randomized into the different treatment groups as above. Mice were then monitored for tumor development, and once the tumor reached a volume of at least 100 mm^3^ (Day 1), the mice were dosed daily for 21 days. The subcutaneous flank tumors were measured three times weekly using calipers. Tumor volume was approximated using the same formula as described for the PC3 model. Mice were weighed daily prior to treatment and assessed for signs of morbidity. The tumors were collected and measured at 21 days after the initiation of treatment. The tissues were subsequently formalin fixed, processed, and stained with hematoxylin and eosin (H&E) and sections were scored in a blinded fashion.

*Pancreatic Cancer Mouse Models*: Six‐ to eight‐week‐old female C57BL/6 mice (Taconic) underwent subcutaneous injection of 1.0 × 10^6^ Panc02‐luc cells into the flank. The mice were randomized into the different groups as above. Mice were monitored for tumor development, and once a tumor reached a volume of at least 100 mm^3^ (Day 1), the mouse was dosed daily for 21 days. The subcutaneous flank tumors were measured three times weekly using calipers. Tumor volume was approximated using the same formula as above. Mice were weighed daily prior to treatment and assessed for signs of morbidity. The tumors were collected and measured at 21 days after the initiation of treatment. The tumor was subsequently formalin fixed, processed, and H&E stained. H&E sections were scored in a blinded fashion.

### Statistical Analyses

The data were presented as means ± SD. Graphs were generated using the GraphPad Prism software. SAS v9.3 was used to conduct all analyses. ANOVA was employed to compare continuous values between three or more groups. For tissue CO quantity analysis, we used a linear mixed effect model fixed effects being the treatments of CO‐GeM or RA‐GeM, and tissue CO content being the response variable. The random effect was individual animal ID to account for individual variability and repeated measures. A significance level of *P* < 0.05 was considered significant.

## Conflict of Interest

HB, DG, LEO, GT, and JDB are co‐inventors on a patent application (WO2022055991A1) submitted by Brigham and Women's Hospital, MIT, and BIDMC that covers therapeutic carbon monoxide formulations. Complete details of all relationships for profit and non for profit for GT can be found at www.dropbox.com/sh/szi7vnr4a2ajb56/AABs5N5i0qAfT1IqIJAE-T5a?dl=0. The authors declare that they have no other competing interests.

## Supporting information

Supporting Information

## Data Availability

The data that support the findings of this study are available from the corresponding author upon reasonable request.
