# Supplementary material for: Oral Carbon Monoxide Enhances Autophagy Modulation in Prostate, Pancreatic, and Lung Cancers
Source: Adv Sci (Weinh). 2023 Dec 12;11(9):2308346. doi: 10.1002/advs.202308346 (PMC10916612; doi:10.1002/advs.202308346)
Supplement: Supplementary file 1 — Supporting Information [file ADVS-11-2308346-s001.pdf]

ADVANCED SCIENCE
Open Access

Supporting Information

for *Adv. Sci.*, DOI 10.1002/advs.202308346

Oral Carbon Monoxide Enhances Autophagy Modulation in Prostate, Pancreatic, and Lung Cancers

*Jianling Bi, Emily Witt, Megan K. McGovern, Arielle B. Cafi, Lauren L. Rosenstock, Anna B. Pearson, Timothy J. Brown, Thomas B. Karasic, Lucas C. Absler, Srija Machkanti, Hannah Boyce, David Gallo, Sarah L. Becker, Keiko Ishida, Joshua Jenkins, Alison Hayward, Alexandra Scheiflinger, Kellie L. Bodeker, Ritesh Kumar, Scott K. Shaw, Salma K. Jabbour, Vitor A. Lira, Michael D. Henry, Michael S. Tift, Leo E. Otterbein, Giovanni Traverso and James D. Byrne**

Supporting Information

Oral carbon monoxide enhances autophagy modulation in prostate, pancreatic, and lung cancers

*Jianling Bi, Emily Witt, Megan K. McGovern, Arielle B. Cafi, Lauren L. Rosenstock, Anna B. Pearson, Timothy J. Brown, Thomas B. Karasic, Lucas C. Absler, Srija Machkanti, Hannah Boyce, David Gallo, Sarah L. Becker, Keiko Ishida, Joshua Jenkins, Alison Hayward, Alexandra Scheiflinger, Kellie L. Bodeker, Ritesh Kumar, Scott K. Shaw, Salma K. Jabbour, Vitor A. Lira, Michael D. Henry, Michael S. Tift, Leo E. Otterbein, Giovanni Traverso, James D. Byrne**

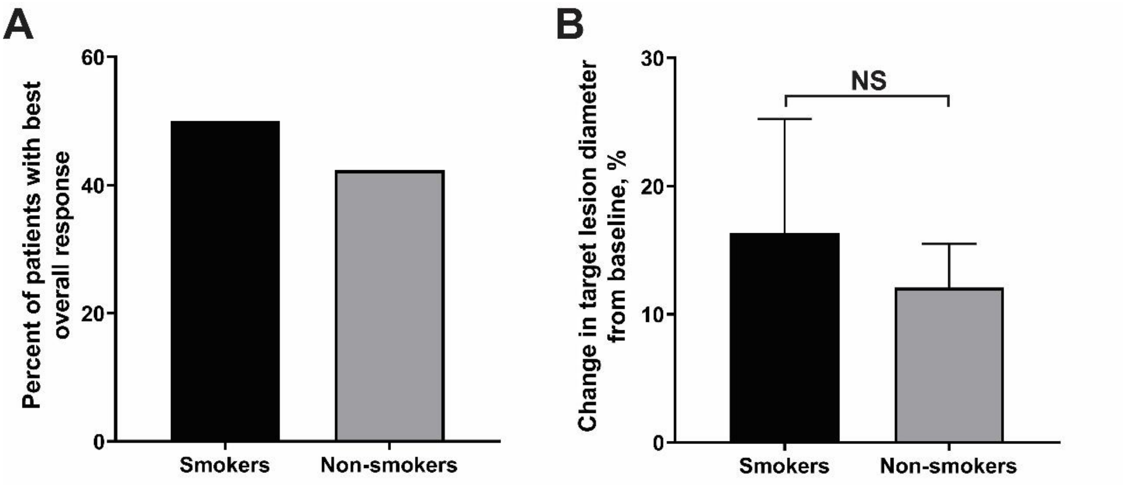

Fig. S1. Active smokers treated with autophagy inhibitors had good outcomes during clinical trial in metastatic lung cancer patients. (**A**) Intention-to-treat analysis for best overall response (OR) in active smokers versus all patients. P values were determined by unpaired t test. NS – not significant. (**B**) Change in diameter of target lesions from baseline, with active smokers identified by each arrow.

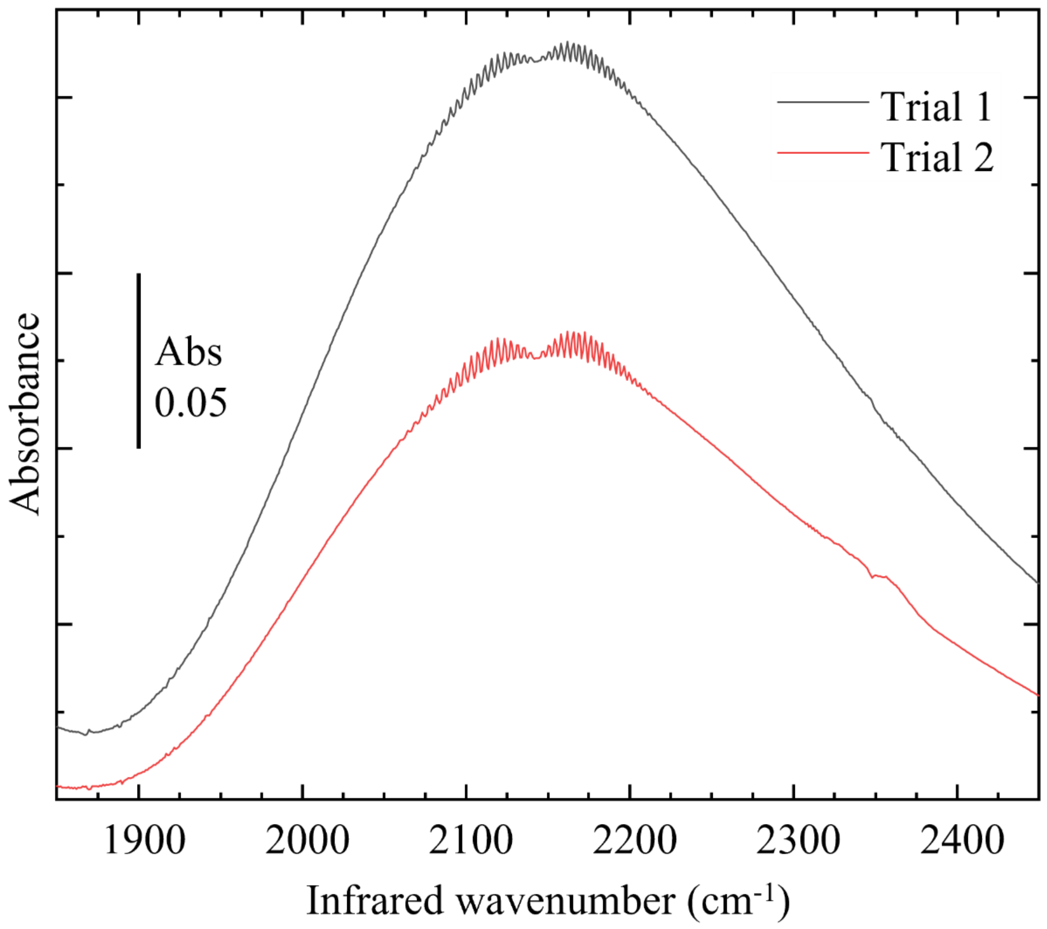

Fig. S2. FTIR spectra for two samples of the CO-GeMs. The CO remains in the gas phase, shown by characteristic oscillations of the R- and P-branches between ca. 2050 cm-1 and 2200 cm-1.

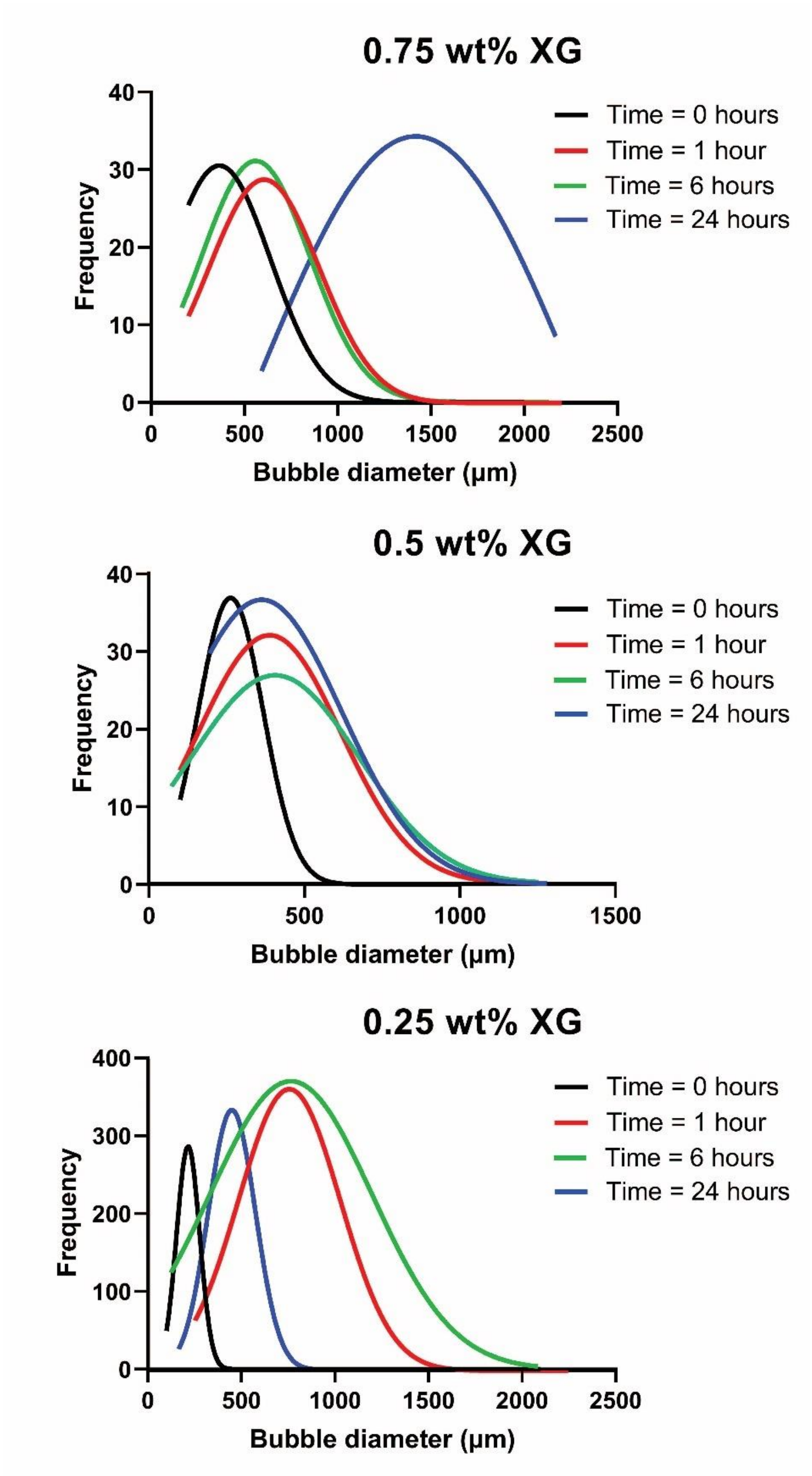

Fig. S3. Gas bubbles increase in size over time in each formulation. Gas bubble size distribution as a function of time. CO-GeMs of 3 different xanthan gum (XG) concentrations were evaluated by Image J analysis of 4x images (n = 10 per time).

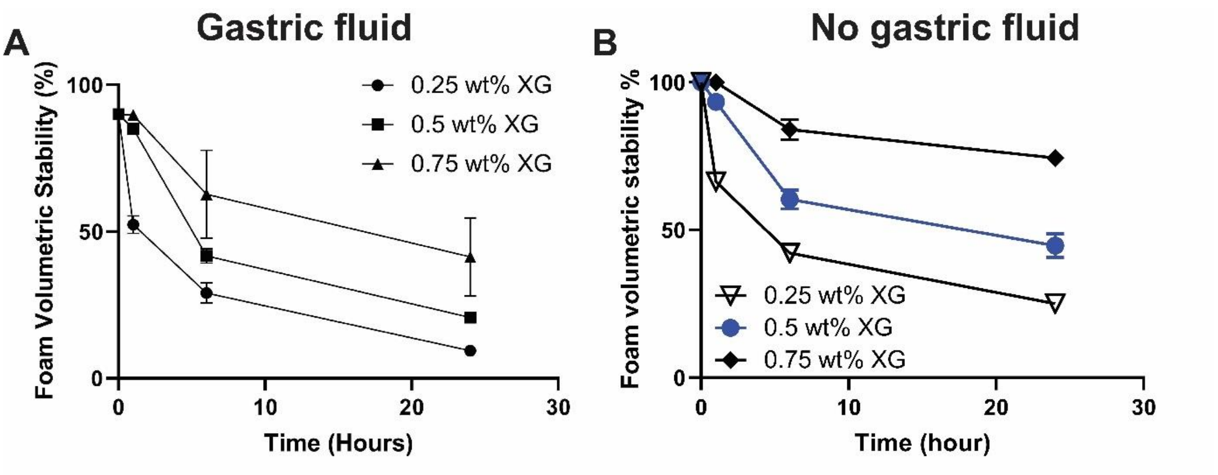

Fig. S4. Volumetric stability of several CO-GeM formulations. Volumetric stability of foam as a function of time for various xanthan gum (XG) concentrations, with maltodextrin held

constant at 0.8 wt % (n = 3 samples per arm). Subfigure (B) was modified with permission from AAAS.

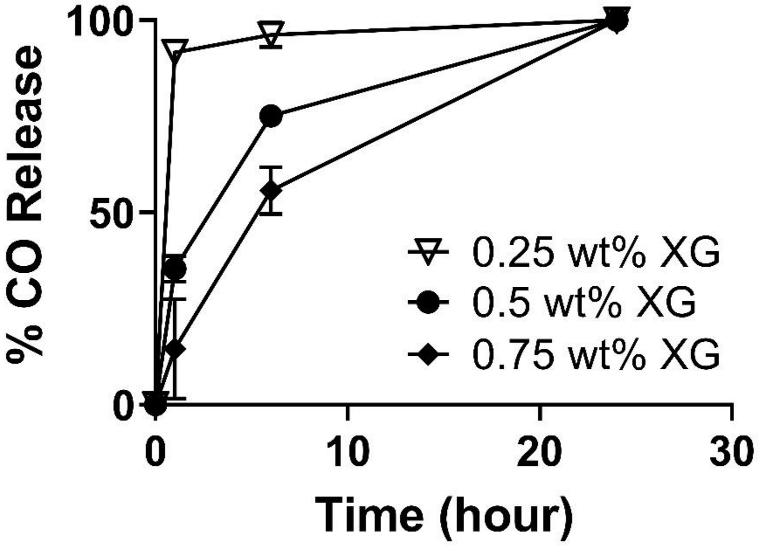

Fig. S5. CO release kinetics from foams with different concentrations of xanthan gum over 24 hours (n = 3 samples per arm).

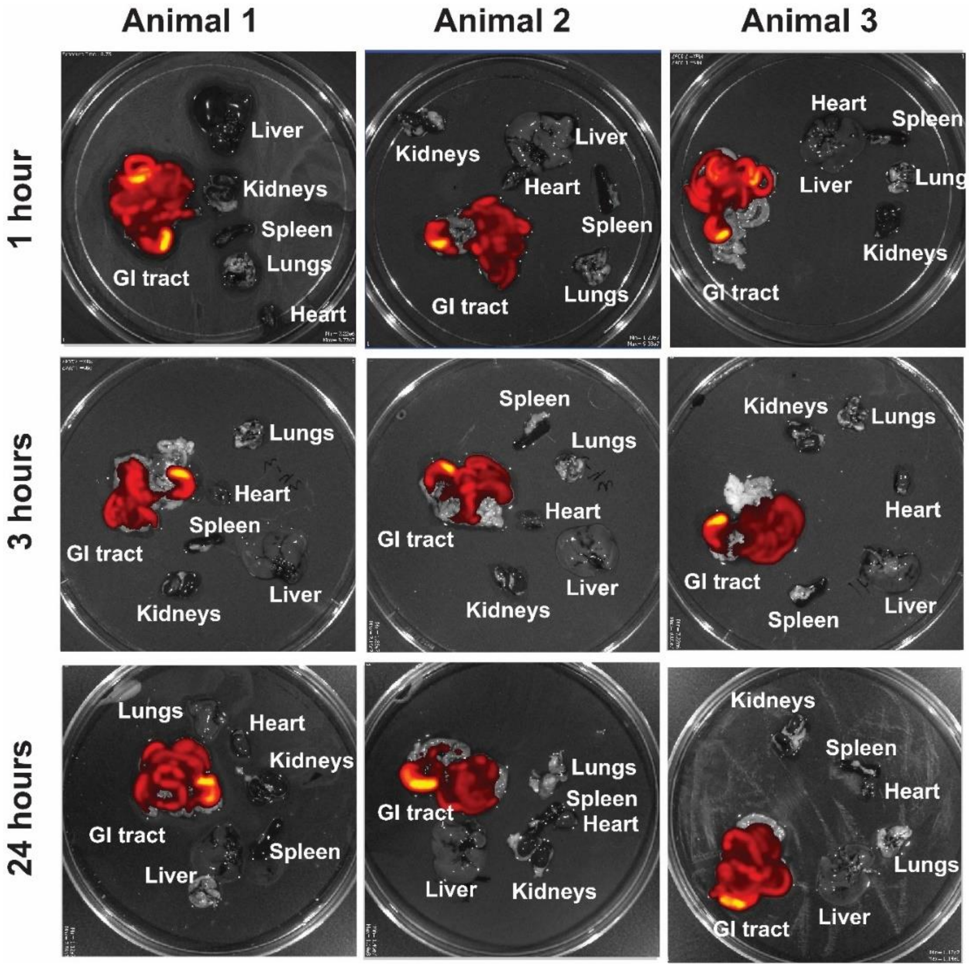

Figure S6. Xanthan gum of the CO-GeMs is retained within the GI tract. Near IR dye-labeled xanthan gum was incorporated into the CO-GeMs to visualize the pharmacokinetics of the non-CO-related components. It was found that the GI tract exclusively after single administration oral gavage (200 µL) with detection for 1, 3, and 24 hours.

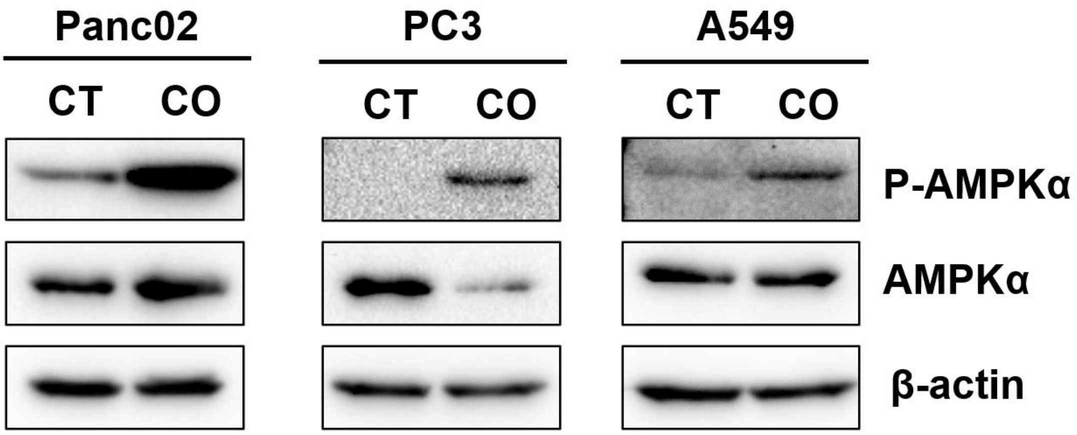

Fig. S7. CO induces autophagy in pancreatic, prostate, and lung cancer cells. Western blotting to assess levels of phosphorylated-AMPKα, LC3, and cleaved caspase 3 in mouse pancreatic cancer cells (Panc02, left), human prostate cancer cells (PC3) and human lung cancer cells (A549, right) exposed to CO or room air.

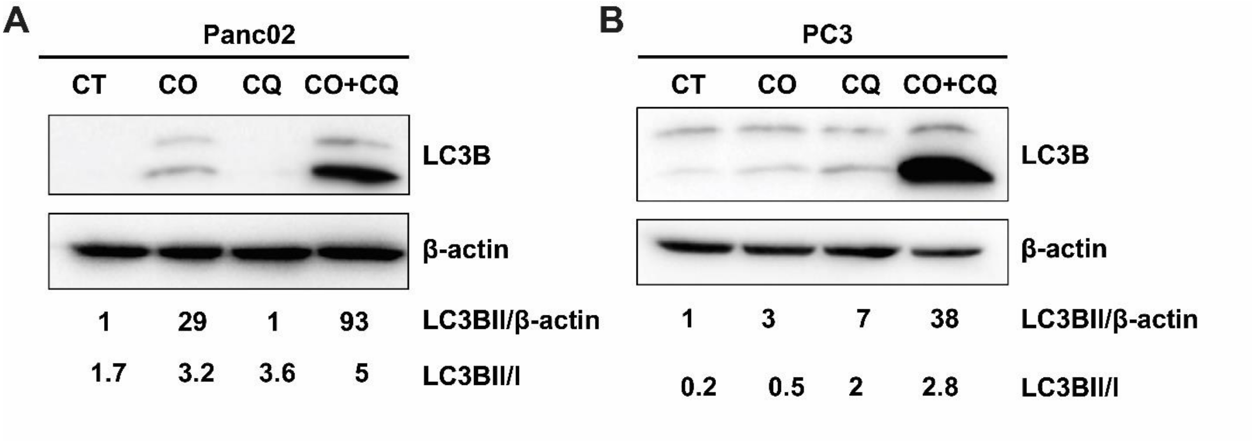

Fig. S8. CO increases LC3BII/I ratio in prostate and pancreatic cancer cells. Western blotting to assess levels of LC3B and β-actin in mouse pancreatic cancer cells (Panc02, left) and human prostate cancer cells (PC3, right) exposed to CO (250 ppm) or room air.

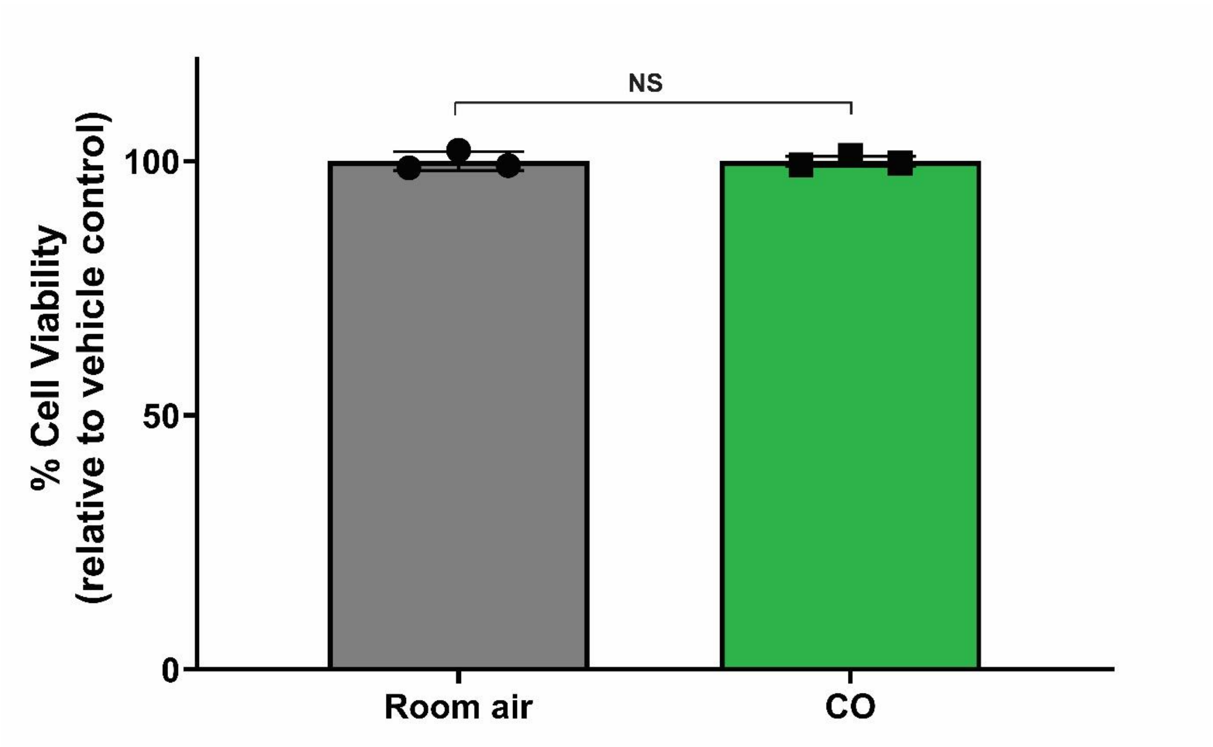

Fig. S9. Exposure of CO to normal intestinal cells does not result in cytotoxicity. Viability of FHs 74 Int cells (human small intestine) were exposed to 250 ppm CO (n = 3). P values were determined by unpaired t test. NS – not significant.

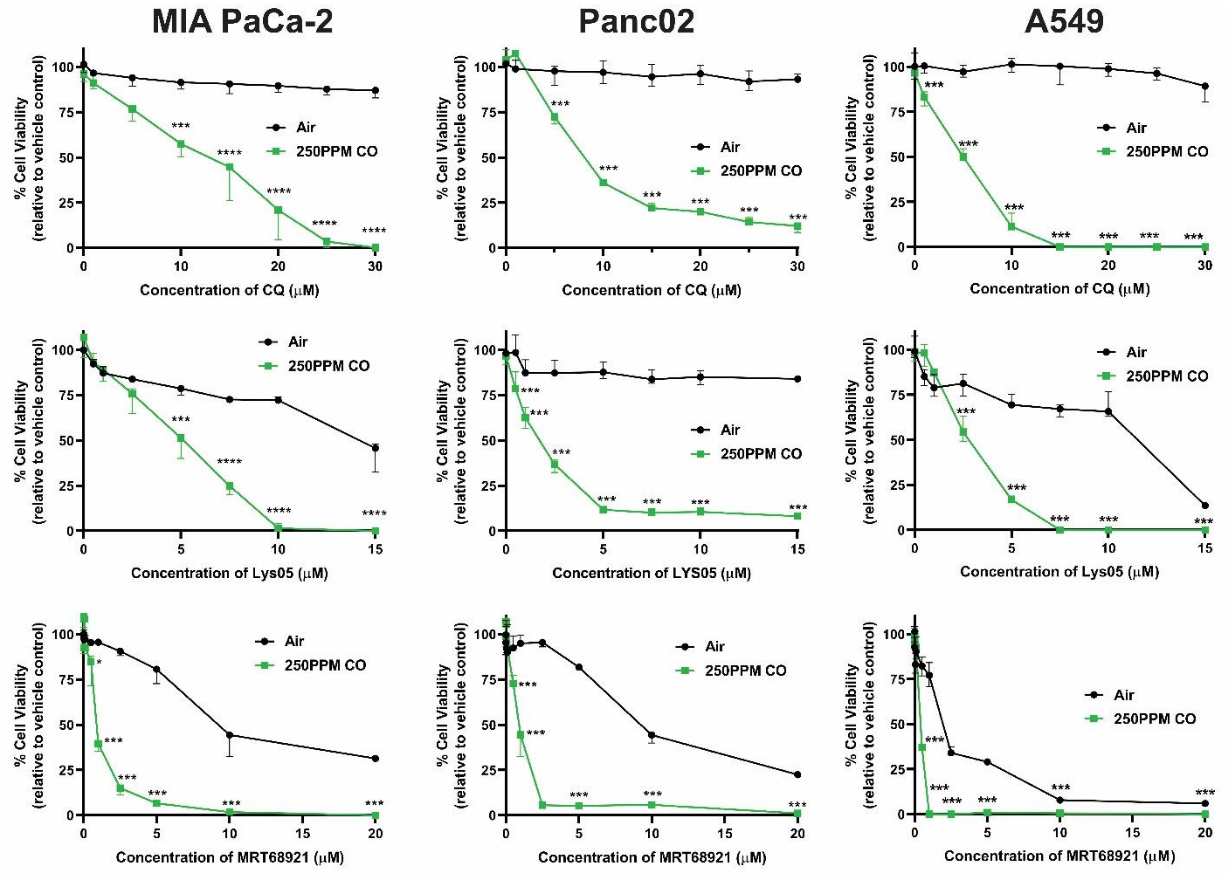

Fig. S10. Concomitant administration of CO and autophagy inhibitors results in increased cancer cell death in pancreatic and lung cancer cell lines. Viability of MIA PaCa-2 (pancreatic), Panc02 (pancreatic), and A549 (lung) cancer cells as a function of the concentration of the autophagy inhibitors chloroquine (CQ), Lys05, and MRT68921, in the absence and presence of CO (n = 8 samples/data point). P values were determined by unpaired t test.

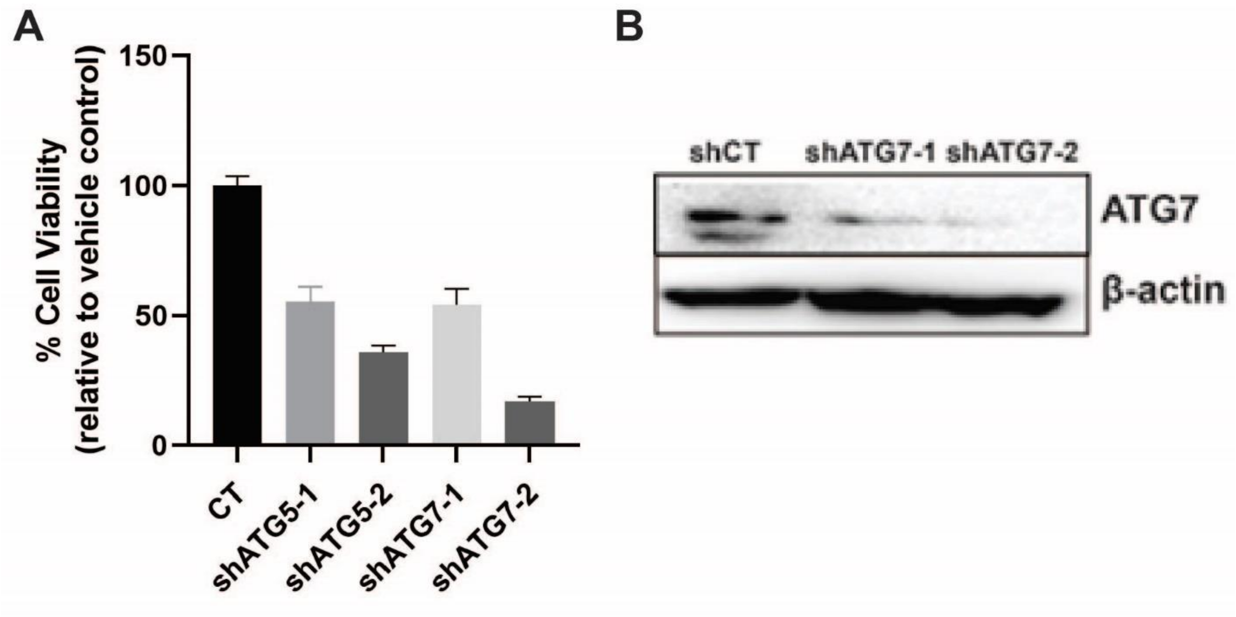

Fig. S11. Effects of shATG-mediated knockdown of ATG5 and ATG7 in PC3 cells. (A) Quantitation of viability of knockdown cells following CO exposure. Controls were non-

transduced cells exposed to 250 ppm CO. (B) Western blotting quantifying levels of ATG7 knockdown in the two lines shown in A.

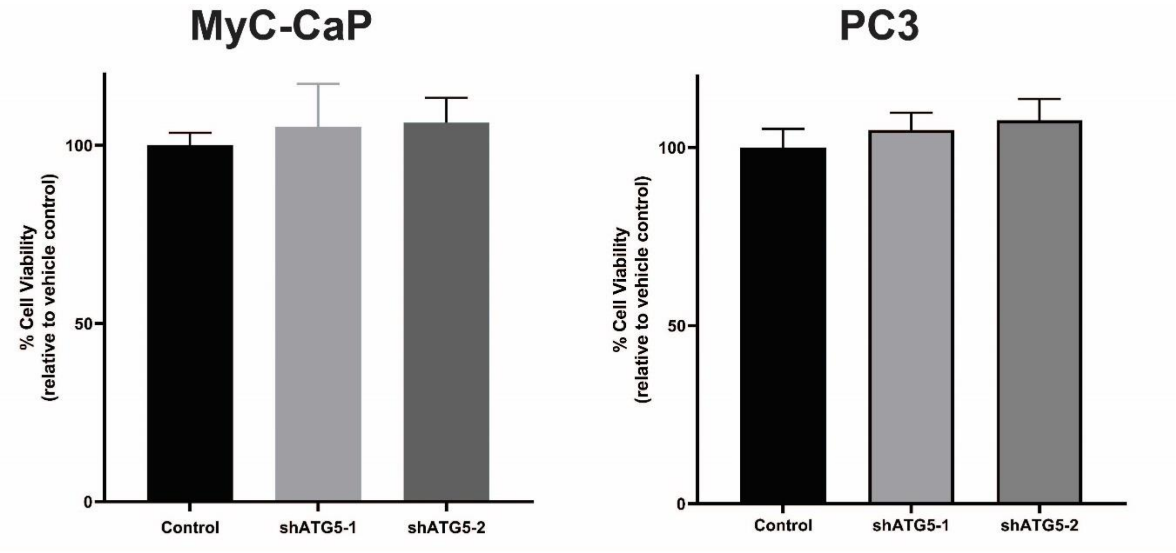

Fig. S12. Effects of shATG-mediated knockdown of ATG5 in room air. Quantitation of viability of knockdown cells following room air exposure. Controls were non-transduced cells exposed to 250 ppm CO.

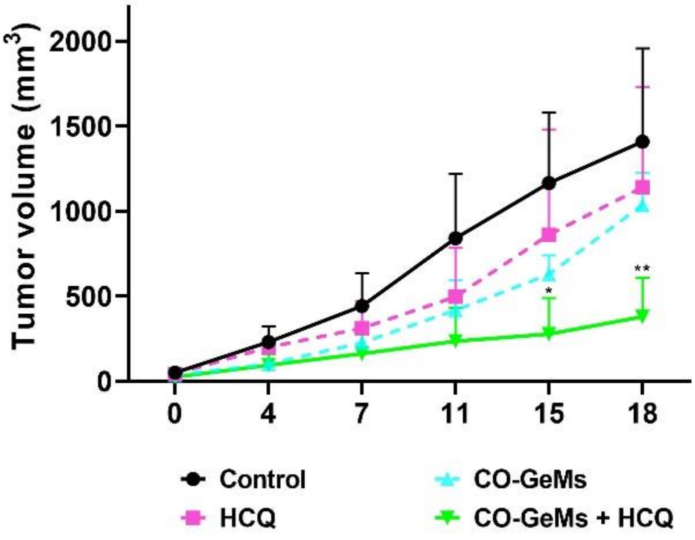

Fig. S13. Anti-tumor effects of CO-GeMs and hydroxychloroquine (HCQ) are enhanced when they are combined. Tumor volume in human pancreatic cancer allografts (Panc02) treated with CO foams and

HCQ, alone and in combination. Data represent means (n = 5-7 mice per arm). P values were determined by unpaired t test.

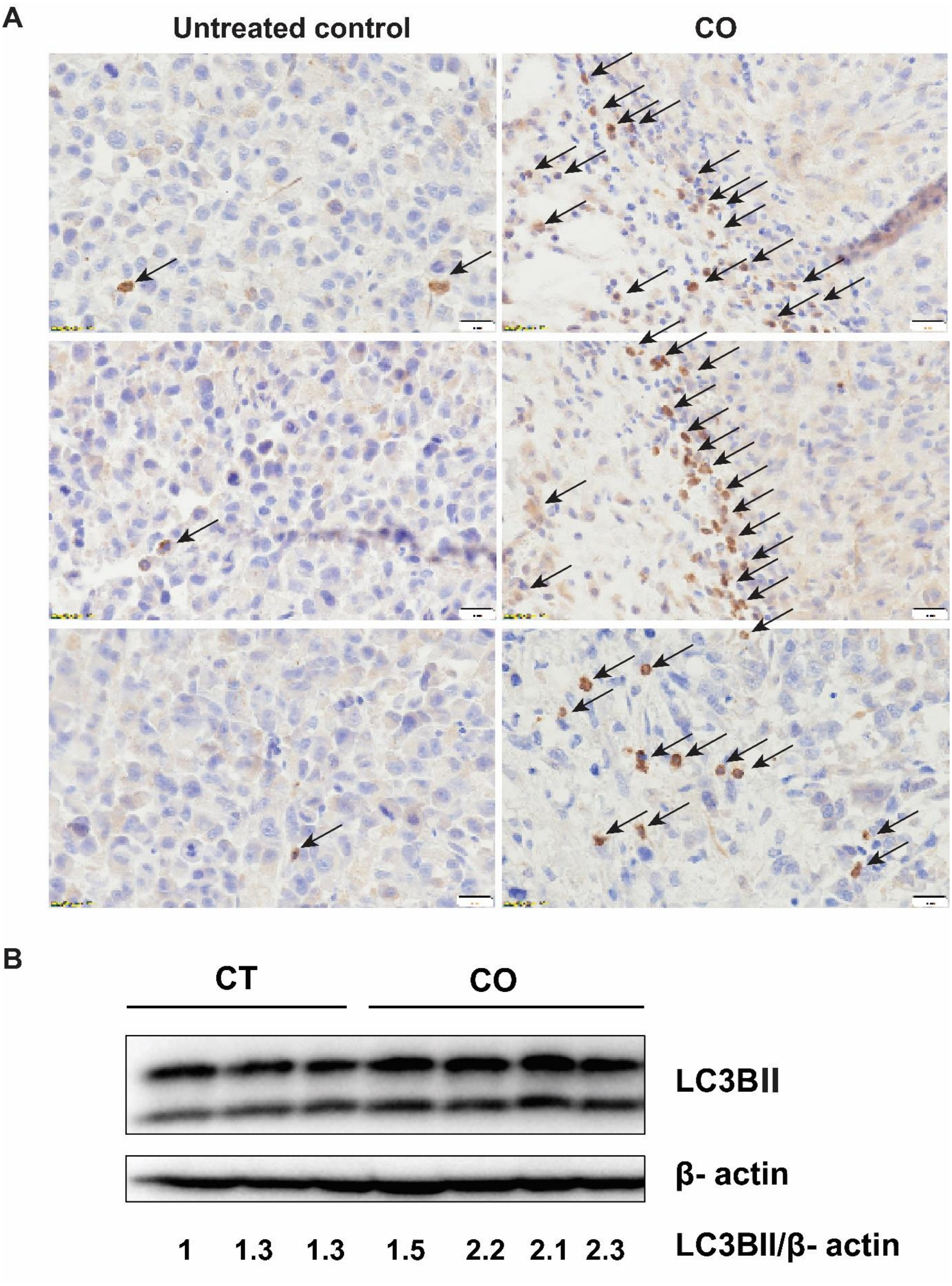
A
Untreated control
CO
B
CT
CO
LC3BII
β- actin
1 1.3 1.3 1.5 2.2 2.1 2.3
LC3BII/β- actin

Fig. S14. Autophagy is induced in tumors in vivo in prostate allograft (MyC-CaP) models exposed to CO-GeMs. Arrows indicate LC3BII staining in tumor tissue, which was further quantified by protein expression as a ratio of LC3BII/β-actin tissue lysates (n = 3-4 tumors/arm).

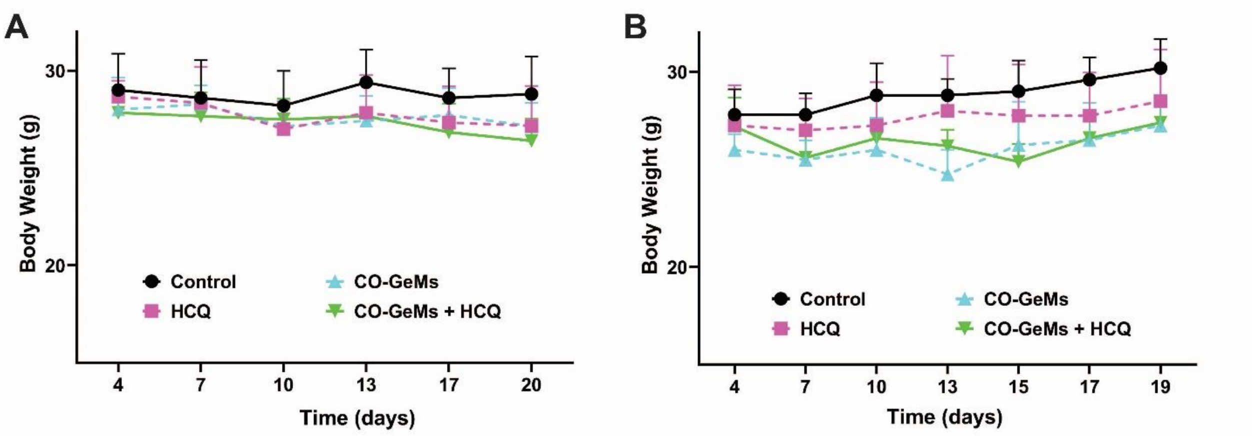

Fig. S15. Weight of mice treated with CO-GeMs, HCQ, or both. Body weight as a function of time in mice bearing PC3 tumors (A) and MyC-CaP tumors (B) treated with HCQ (hydroxychloroquine), CO-GeMs, CO-GeMs + HCQ, or no treatment.

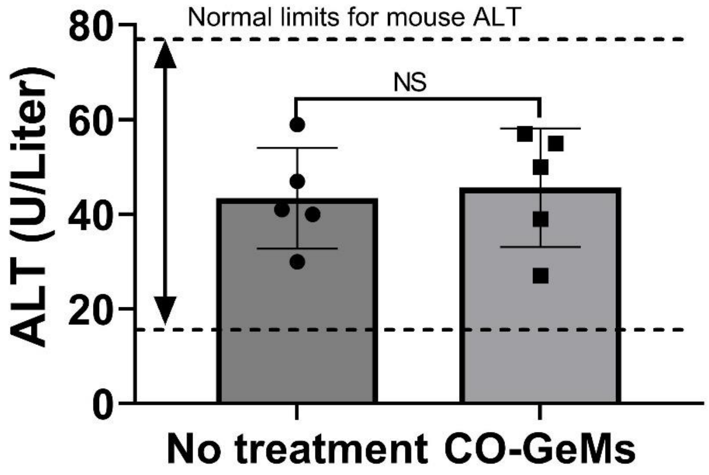

Fig. S16. CO-GeMs do not induce hepatotoxicity at current dosing. ALT (U/L) as measured in mouse plasma from mice treated with three doses of CO-GeMs (200 µL) via oral gavage compared to untreated mice (n = 5 per arm). P value was obtained using unpaired t test.

Table S1. Results from clinical trials of autophagy inhibitors in patients with solid tumors.

Cancer type	# of patients	Phase	mOS (months)	ORR	mPFS (months)	Ref
Advanced solid tumors	35	I	NR	NR	NR	[19]
Advanced solid tumors	24	I	NR	NR	NR	[4d]
Advanced solid tumors	27	I	NR	74%	3.5	[4m]
Advanced solid tumors	27	I	NR	11%	NR	[4k]
CRC	20	I	5.2	1%	1.8	[4i]
CRC	42	II	7.23	NR	1.9	[4b]
GBM	92	I/II	15.6	NR	NR	[4n]
Melanoma	34	I/II	NR	Total: 85%<br>Elevated LDH: 88%	11.2	[4a]
Metastatic CRC	38	II	25.9	75%	13.9	[20]
NSCLC	40	Ib/II	NR	Total: 33%<br>KRAS(+): 44%	Total: 3.3<br>KRAS(+): 6.4	[4l]

Pancreatic	34	II	34.83	NR	NR	[4p]
Pancreatic	112	II	At 12 months<br>HCQ: 41%<br>non-HCQ: 51%	HCQ: 38.2%<br>non-HCQ: 21.1%	HCQ: 5.7<br>non-HCQ: 6.4	[4j]
Pancreatic	35	I/II	LC3-II increase<br>> 51%: 34.83<br>≤ 51%: 10.83	NR	LC3-II increase<br>> 51%: 15.03<br>≤ 51%: 6.9	[4c]
Pancreatic	20	II	2.3	10%	1.5	[4o]
Prostate	21	II	15.7	NR	5.3	[4h]
Prostate	64		NR	NR	NR	[4f]
RCC	38	I/II	NR	67%	45% ≥ 6 mos	[4g]
Sarcoma	10	II	NR	60%	NR	[4e]

NR = Not reported, HCQ = hydroxychloroquine

References

[1] Y. Aman, T. Schmauck-Medina, M. Hansen, R. I. Morimoto, A. K. Simon, I. Bjedov, K. Palikaras, A. Simonsen, T. Johansen, N. Tavernarakis, D. C. Rubinsztein, L. Partridge, G. Kroemer, J. Labbadia, E. F. Fang, *Nat Aging* **2021**, *1* (8), 634, https://doi.org/10.1038/s43587-021-00098-4.

[2] N. Mizushima, *Nat Cell Biol* **2018**, *20* (5), 521, https://doi.org/10.1038/s41556-018-0092-5.

[3] J. M. M. Levy, C. G. Towers, A. Thorburn, *Nat Rev Cancer* **2017**, *17* (9), 528, https://doi.org/10.1038/nrc.2017.53.

[4] a) R. K. Amaravadi, *Autophagy* **2022**, *18* (6), 1470, https://doi.org/10.1080/15548627.2022.2038899; b) S. P. Arora, L. Tenner, J. Sarantopoulos, J. Morris, Q. Liu, J. A. Mendez, T. Curiel, J. Michalek, D. Mahalingam, *Br J Cancer* **2022**, *127* (6), 1153, https://doi.org/10.1038/s41416-022-01892-6; c) B. A. Boone, N. Bahary, A. H. Zureikat, A. J. Moser, D. P. Normolle, W. C. Wu, A. D. Singhi, P. Bao, D. L. Bartlett, L. A. Liotta, V. Espina, P. Loughran, M. T. Lotze, H. J. Zeh, 3rd, *Ann Surg Oncol* **2015**, *22* (13), 4402, https://doi.org/10.1245/s10434-015-4566-4; d) I. Brana, A. Ocana, E. X. Chen, A. R. Razak, C. Haines, C. Lee, S. Douglas, L. Wang, L. L. Siu, I. F. Tannock, P. L. Bedard, *Invest New Drugs* **2014**, *32* (6), 1269, https://doi.org/10.1007/s10637-014-0159-5; e) M. S. Chi, C. Y. Lee, S. C. Huang, K. L. Yang, H. L. Ko, Y. K. Chen, C. H. Chung, K. W. Liao, K. H. Chi, *Oncotarget* **2015**, *6* (30), 29808, https://doi.org/10.18632/oncotarget.5060; f) M. A. George, T. M. Mayer, D. Moore, C. Chen, E. White, R. S. DiPaola, C. Jeyamohan, M. N. Stein, *Journal of Clinical Oncology* **2017**, *35* (6_suppl); g) N. B. Haas, L. J. Appleman, M. Stein, M. Redlinger, M. Wilks, X. Xu, A. Onorati, A. Kalavacharla, T. Kim, C. J. Zhen, S. Kadri, J. P. Segal, P. A. Gimotty, L. E. Davis, R. K. Amaravadi, *Clin Cancer Res* **2019**, *25* (7), 2080, https://doi.org/10.1158/1078-0432.CCR-18-2204; h) A. R. Hansen, I. F. Tannock, A. Templeton, E. Chen, A. Evans, J. Knox, A. Prawira, S. S. Sridhar, S. Tan, F. Vera-Badillo, L. Wang, B. G. Wouters, A. M. Joshua, *Oncologist* **2019**, *24* (9), 1188, https://doi.org/10.1634/theoncologist.2018-0621; i) T. B. Karasic, T. J. Brown, C. Schneider, U. R. Teitelbaum, K. A. Reiss, T. C. Mitchell, R. C. Massa, M. H. O'Hara, L. DiCicco, L. Garcia-Marcano, R. K. Amaravadi, P. J. O'Dwyer, *Oncologist* **2022**, *27* (9), 716, https://doi.org/10.1093/oncolo/oyac078; j) T. B. Karasic, M. H. O'Hara, A. Loaiza-Bonilla, K. A. Reiss, U. R. Teitelbaum, E. Borazanci, A. De Jesus-Acosta, C. Redlinger, J. A. Burrell, D. A. Laheru, D. D. Von Hoff, R. K. Amaravadi, J. A. Drebin, P. J. O'Dwyer, *JAMA Oncol* **2019**, *5* (7), 993, https://doi.org/10.1001/jamaoncol.2019.0684; k) D. Mahalingam, M. Mita, J. Sarantopoulos, L. Wood, R. K. Amaravadi, L. E. Davis, A. C. Mita, T. J. Curiel, C. M. Espitia, S. T. Nawrocki, F. J. Giles, J. S. Carew, *Autophagy* **2014**, *10* (8), 1403, https://doi.org/10.4161/auto.29231; l) J. Malhotra, S. Jabbour, M. Orlick, G. Riedlinger, Y. Guo, E. White, J. Aisner, *Cancer Treat Res Commun* **2019**, *21*, 100158, https://doi.org/10.1016/j.ctarc.2019.100158; m) R. Rangwala, Y. C. Chang, J. Hu, K. M. Algazy, T. L. Evans, L. A. Fecher, L. M. Schuchter, D. A. Torigian, J. T. Panosian, A. B. Troxel, K. S. Tan, D. F. Heitjan, A. M. DeMichele, D. J. Vaughn, M. Redlinger, A. Alavi, J. Kaiser, L. Pontiggia, L. E. Davis, P. J. O'Dwyer, R. K. Amaravadi, *Autophagy* **2014**, *10* (8), 1391, https://doi.org/10.4161/auto.29119; n) M. R. Rosenfeld, X. Ye, J. G. Supko, S. Desideri, S. A. Grossman, S. Brem, T. Mikkelson, D. Wang, Y. C. Chang, J. Hu, Q. McAfee, J. Fisher, A. B. Troxel, S. Piao, D. F. Heitjan, K. S. Tan, L. Pontiggia, P. J. O'Dwyer, L. E.

Davis, R. K. Amaravadi, *Autophagy* **2014**, *10* (8), 1359, https://doi.org/10.4161/auto.28984; o) B. M. Wolpin, D. A. Rubinson, X. Wang, J. A. Chan, J. M. Cleary, P. C. Enzinger, C. S. Fuchs, N. J. McCleary, J. A. Meyerhardt, K. Ng, D. Schrag, A. L. Sikora, B. A. Spicer, L. Killion, H. Mamon, A. C. Kimmelman, *Oncologist* **2014**, *19* (6), 637, https://doi.org/10.1634/theoncologist.2014-0086; p) H. J. Zeh, N. Bahary, B. A. Boone, A. D. Singhi, J. L. Miller-Ocuin, D. P. Normolle, A. H. Zureikat, M. E. Hogg, D. L. Bartlett, K. K. Lee, A. Tsung, J. W. Marsh, P. Murthy, D. Tang, N. Seiser, R. K. Amaravadi, V. Espina, L. Liotta, M. T. Lotze, *Clin Cancer Res* **2020**, *26* (13), 3126, https://doi.org/10.1158/1078-0432.CCR-19-4042.

[5] T. Liu, J. Zhang, K. Li, L. Deng, H. Wang, *Front Pharmacol* **2020**, *11*, 408, https://doi.org/10.3389/fphar.2020.00408.

[6] a) A. Aroca, C. Gotor, *Antioxidants (Basel)* **2022**, *11* (2), https://doi.org/10.3390/antiox11020327; b) S. Sarkar, V. I. Korolchuk, M. Renna, S. Imarisio, A. Fleming, A. Williams, M. Garcia-Arencibia, C. Rose, S. Luo, B. R. Underwood, G. Kroemer, C. J. O'Kane, D. C. Rubinsztein, *Mol Cell* **2011**, *43* (1), 19, https://doi.org/10.1016/j.molcel.2011.04.029; c) S. J. Lee, S. W. Ryter, J. F. Xu, K. Nakahira, H. P. Kim, A. M. Choi, Y. S. Kim, *Am J Respir Cell Mol Biol* **2011**, *45* (4), 867, https://doi.org/10.1165/rcmb.2010-0352OC.

[7] a) P. Chakraborty, R. Y. Parikh, S. Choi, D. Tran, M. Gooz, Z. T. Hedley, D. S. Kim, D. Pytel, I. Kang, S. N. Nadig, G. C. Beeson, L. Ball, M. Mehrotra, H. Wang, S. Berto, V. Palanisamy, H. Li, S. Chatterjee, P. C. Rodriguez, E. N. Maldonado, J. A. Diehl, V. K. Gangaraju, S. Mehrotra, *Cancer Res* **2022**, *82* (10), 1969, https://doi.org/10.1158/0008-5472.CAN-21-3155; b) H. J. Kim, Y. Joe, S. Y. Rah, S. K. Kim, S. U. Park, J. Park, J. Kim, J. Ryu, G. J. Cho, Y. J. Surh, S. W. Ryter, U. H. Kim, H. T. Chung, *Cell Death Dis* **2018**, *9* (11), 1060, https://doi.org/10.1038/s41419-018-1112-x.

[8] J. D. Byrne, D. Gallo, H. Boyce, S. L. Becker, K. M. Kezar, A. T. Cotoia, V. R. Feig, A. Lopes, E. Csizmadia, M. S. Longhi, J. S. Lee, H. Kim, A. J. Wentworth, S. Shankar, G. R. Lee, J. Bi, E. Witt, K. Ishida, A. Hayward, J. L. P. Kuosmanen, J. Jenkins, J. Wainer, A. Aragon, K. Wong, C. Steiger, W. R. Jeck, D. E. Bosch, M. C. Coleman, D. R. Spitz, M. Tift, R. Langer, L. E. Otterbein, G. Traverso, *Sci Transl Med* **2022**, *14* (651), eabl4135, https://doi.org/10.1126/scitranslmed.abl4135.

[9] J. Bi, E. Witt, V. A. Voltarelli, V. R. Feig, V. Venkatachalam, H. Boyce, M. McGovern, W. R. Gutierrez, J. D. Rytlewski, K. R. Bowman, A. C. Rhodes, A. N. Cook, B. N. Muller, M. G. Smith, A. R. Ramos, H. Panchal, R. D. Dodd, M. D. Henry, A. Mailloux, G. Traverso, L. E. Otterbein, J. D. Byrne, *Adv Sci (Weinh)* **2023**, e2205995, https://doi.org/10.1002/advs.202205995.

[10] J. D. Belcher, E. Gomperts, J. Nguyen, C. Chen, F. Abdulla, Z. M. Kiser, D. Gallo, H. Levy, L. E. Otterbein, G. M. Vercellotti, *PLoS One* **2018**, *13* (10), e0205194, https://doi.org/10.1371/journal.pone.0205194.

[11] a) F. Hua, K. Li, J. J. Yu, X. X. Lv, J. Yan, X. W. Zhang, W. Sun, H. Lin, S. Shang, F. Wang, B. Cui, R. Mu, B. Huang, J. D. Jiang, Z. W. Hu, *Nat Commun* **2015**, *6*, 7951, https://doi.org/10.1038/ncomms8951; b) N. Hariharan, P. Zhai, J. Sadoshima, *Antioxid Redox Signal* **2011**, *14* (11), 2179, https://doi.org/10.1089/ars.2010.3488.

[12] L. Cui, L. P. Zhao, J. Y. Ye, L. Yang, Y. Huang, X. P. Jiang, Q. Zhang, J. Z. Jia, D. X. Zhang, Y. Huang, *Front Cell Dev Biol* **2020**, *8*, 31, https://doi.org/10.3389/fcell.2020.00031.

[13] Q. Liu, M. Zhao, W. Chen, K. Xu, F. Huang, J. Qu, Z. Xu, X. Wang, Y. Wang, Y. Zhu, W. Wang, *Arch Oral Biol* **2020**, *111*, 104646, https://doi.org/10.1016/j.archoralbio.2019.104646.

[14] Y. Zhou, W. Yu, J. Cao, H. Gao, *Biomaterials* **2020**, *255*, 120193, https://doi.org/10.1016/j.biomaterials.2020.120193.

[15] B. Wegiel, D. Gallo, E. Csizmadia, C. Harris, J. Belcher, G. M. Vercellotti, N. Penacho, P. Seth, V. Sukhatme, A. Ahmed, P. P. Pandolfi, L. Helczynski, A. Bjartell, J. L. Persson, L. E. Otterbein, *Cancer Res* **2013**, *73* (23), 7009, https://doi.org/10.1158/0008-5472.CAN-13-1075.

[16] a) L. E. Fredenburgh, M. A. Perrella, D. Barragan-Bradford, D. R. Hess, E. Peters, K. E. Welty-Wolf, B. D. Kraft, R. S. Harris, R. Maurer, K. Nakahira, C. Oromendia, J. D. Davies, A. Higuera, K. T. Schiffer, J. A. Englert, P. B. Dieffenbach, D. A. Berlin, S. Lagambina, M. Bouthot, A. I. Sullivan, P. F. Nuccio, M. T. Kone, M. J. Malik, M. A. P. Porras, E. Finkelsztein, T. Winkler, S. Hurwitz, C. N. Serhan, C. A. Piantadosi, R. M. Baron, B. T. Thompson, A. M. Choi, *JCI Insight* **2018**, *3* (23), https://doi.org/10.1172/jci.insight.124039; b) I. O. Rosas, H. J. Goldberg, H. R. Collard, S. El-Chemaly, K. Flaherty, G. M. Hunninghake, J. A. Lasky, D. J. Lederer, R. Machado, F. J. Martinez, R. Maurer, D. Teller, I. Noth, E. Peters, G. Raghu, J. G. N. Garcia, A. M. K. Choi, *Chest* **2018**, *153* (1), 94, https://doi.org/10.1016/j.chest.2017.09.052; c) E. Bathoorn, D. J. Slebos, D. S. Postma, G. H. Koeter, A. J. van Oosterhout, M. van der Toorn, H. M. Boezen, H. A. Kerstjens, *Eur Respir J* **2007**, *30* (6), 1131, https://doi.org/10.1183/09031936.00163206.

[17] W. Zhang, W. Fan, S. Rachagani, Z. Zhou, S. M. Lele, S. K. Batra, J. C. Garrison, *Sci Rep* **2019**, *9* (1), 11117, https://doi.org/10.1038/s41598-019-47308-z.

[18] a) H. J. Vreman, L. K. Kwong, D. K. Stevenson, *Clin Chem* **1984**, *30* (8), 1382; b) H. J. Vreman, R. J. Wong, T. Kadotani, D. K. Stevenson, *Anal Biochem* **2005**, *341* (2), 280, https://doi.org/10.1016/j.ab.2005.03.019.

[19] J. M. Mehnert, A. D. Kaveney, J. Malhotra, K. Spencer, D. Portal, S. Goodin, A. R. Tan, J. Aisner, R. A. Moss, H. Lin, J. R. Bertino, D. Gibbon, L. A. Doyle, E. P. White, M. N. Stein, *Cancer Chemother Pharmacol* **2019**, *84* (4), 899, https://doi.org/10.1007/s00280-019-03919-x.

[20] E. Poplin, Hydroxychloroquine, capecitabine, oxaliplatin, and bevacizumab in treating patients with metastatic colorectal cancer, clinicaltrials.gov/ct2/show/results/NCT01006369, accessed.
